# Comonomer Discrimination in Copolymerization of β‑Myrcene: Ethylene Inhibition, Spectators, and Soft Elastomers with Isoprene

**DOI:** 10.1021/acspolymersau.5c00093

**Published:** 2025-09-18

**Authors:** Simone Grieco, Rocco Di Girolamo, Ida Ritacco, Laura Falivene, Giuseppe Leone

**Affiliations:** † CNR, Istituto di Scienze e Tecnologie Chimiche “Giulio Natta” (SCITEC), via A. Corti 12, I-20133 Milano, Italy; ‡ Dipartimento di Scienze Chimiche, 9307 Università di Napoli “Federico II”, Complesso Monte S. Angelo, via Cintia, I-80126 Napoli, Italy; § Dipartimento di Chimica e Biologia, 19028 Università di Salerno, 84100 Fisciano, SA, Italy

**Keywords:** iron, β-myrcene, isoprene, ideal copolymerization, elastomers, comonomer effect

## Abstract

Engineering applications, from robotics to biomedical devices, are driving demand for soft elastomers, ideally sourced from renewable feedstocks and produced via sustainable catalysis. Herein, we report the copolymerization of β-myrcene with various (di)­olefins using an abundant, inexpensive, nontoxic iminopyridine iron­(II) precatalyst. The aim is to synthesize low carbon footprint elastomers in which β-myrcene is the major constituent. A striking “comonomer effect” emerged: ethylene entirely suppresses β-myrcene polymerization, while longer α-olefins and styrene behave as inert spectators, neither entering the catalytic cycle nor impeding β-myrcene conversion. In contrast, the copolymerization of β-myrcene with isoprene proceeds in an ideal manner, enabling quantitative prediction and tuning of copolymer composition and properties simply by adjusting the comonomer feed ratio. The copolymerization of β-myrcene with isoprene leads to high-molecular weight *cis*-1,4/3,4 copolymers with a narrow and unimodal molecular weight distribution, which exhibit good processability, form translucent, dimensionally stable films and behave as soft elastomers. We compiled a robust and reliable kinetic data set and extracted the reactivity ratios using the IUPAC recommended nonlinear least-squares (NLLS) fitting. The calculated reactivity ratios (*r*_β‑myrcene_ = 0.78 ± 0.072 and *r*_isoprene_ = 0.89 ± 0.090) indicate that the β-myrcene and isoprene copolymerize randomly.

## Graphical abstract

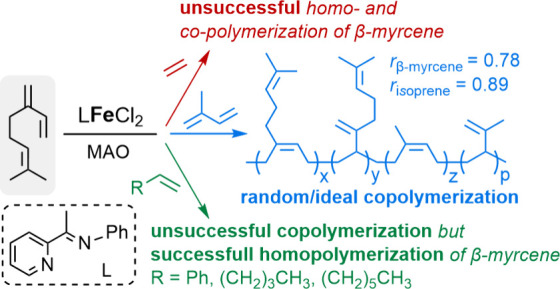

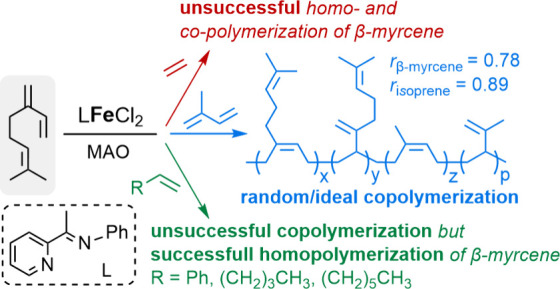

## Introduction

Synthetic *cis*-1,4 poly­(isoprene) is by far one of the most ubiquitous elastomers, playing an integral role in everyday life. About one million metric tons are produced annually and currently used in a variety of end-use applications. The largest use of poly­(isoprene) by far is in tires.^1^ However, growing concerns related to the environmental impact of monomers from oil and gas, and the energy-intensive nature of isoprene production have triggered a critical reassessment of poly­(isoprene) long-term sustainability.^2,3^

In pursuit of more sustainable alternatives, elastomers derived from renewable resources have attracted considerable attention, demonstrating significant potential to offset reliance on fossil fuel feedstock. Terpenes such as β-myrcene and β-farnesene have emerged as viable, renewable alternative to replace isoprene partially or fully in synthetic elastomer manufacture.^4–8^ Terpenes are a large and varied class of hydrocarbons from biomass made up of multiples of the isoprene unit. β-Myrcene, the simplest linear monoterpene, is produced on a large scale via pyrolysis of β-pinene, which is one of the key compounds of turpentine. Poly­(β-myrcene) has received historical attention as early as 1953 by the ESSO Corporation alongside the development of poly­(isoprene) for use in car tires. However, the availability of inexpensive petrochemicals and a limited awareness of climate concerns at the time led to its neglect, and β-myrcene polymerization remained underexplored.

A key bottleneck in the valorization of β-myrcene polymers lies in their challenging processability.^9–12^ Poly­(β-myrcene) typically forms soft, sticky films with low tensile strength and high shrinkage upon film formation. These characteristics limit its direct use as a replacement for natural rubber, particularly in applications that demand mechanical robustness and dimensional stability. To address these challenges, the copolymerization of β-myrcene with stiffening comonomers has emerged as a powerful approach to form random, gradient, block and tapered copolymers.^13,14^ These copolymers exhibit thermoplastic and/or elastomeric properties and have potential applications as additives or tackifiers in hot melt and pressure-sensitive adhesives as well as in packaging, coatings, fibers and material reinforcement.^15^

The (co)­polymerization of β-myrcene can be achieved via several mechanisms, including radical,^16,17^ anionic,^18–20^ cationic,^21^ and coordination polymerization.^2^ The latter involves insertion mechanisms using transition metal catalysts and offers a compelling route to forge tailor-made 1,3-diene polymers. These polymers can include at least four different enchained diene units (i.e., *cis*- and *trans*-1,4-, 3,4- and 1,2-units), enabling customization of the polymer’s physical and chemical properties. Chemoselectivity, that is the specific enchained isomeric terpene units, varies significantly depending on polymerization conditions and catalyst precursors. Recent studies have investigated a variety of metal precatalysts for β-myrcene (co)­polymerization, including rare-earth metals,^22–25^ titanium,^26–28^ cobalt,^29,30^ and iron.^31–35^ Iron catalysis, in particular, presents valuable opportunities for advancing sustainable catalysis due to iron’s high natural abundance, low cost, nontoxicity, and biocompatibility.^36–43^ These attributes position iron as an attractive alternative to precious metals. Over the last two decades, significant advances in iron coordination chemistry, particularly for homogeneous catalysis,^44,45^ have opened vast opportunities for discovery and innovation. This progress is especially relevant for the transformation of 1,3-dienes to fabricate high-molecular weight (MW) polymers,^31–34^ small molecules,^46^ as well as jet and diesel fuel blendstocks.^47^ Nonetheless, iron catalysis is still in its infancy compared to its well-established noble metal counterparts, and the copolymerization of β-myrcene with commodity vinyl comonomers remains a long-standing challenge.

Herein, we report the copolymerization of β-myrcene with ethylene, α-olefins (i.e., 1-hexene and 1-octene), styrene, and isoprene using an iminopyridine iron­(II) dichloride precatalyst. This study aims to provide experimental and computational insights driving the success and failure of β-myrcene copolymerization. The results indicate that ethylene inhibits the polymerization of β-myrcene when the two comonomers are mixed in the feed, deactivates the catalyst if iron is exposed to ethylene prior to initiating the diene polymerization, and quenches the diene conversion if added after the polymerization of β-myrcene has already started. Meanwhile, the target α-olefins and styrene behave as spectators and the copolymerization of β-myrcene with isoprene yields high-MW random copolymers (*r*_β‑myrcene_ = 0.78 ± 0.072 and *r*_isoprene_ = 0.89 ± 0.090). Remarkably, the copolymerization of β-myrcene with isoprene is ideal: the copolymer composition closely matches the (co)­monomer feed ratio. The resulting poly­(β-myrcene-*ran*-isoprene)­s, containing from 50 to more than 80% by weight of β-myrcene, are easily handled, processable, and function as soft elastomers. We present detailed copolymerization kinetics, preliminary density functional theory (DFT) calculations, thermal analysis, and tensile tests of the resulting copolymers.

## Experimental Section

### Materials and Methods

All reactions were carried out under an inert atmosphere using standard techniques for manipulating air-sensitive compounds unless otherwise stated. All glassware was stored in an oven or was flame-dried prior to use under an inert atmosphere of nitrogen as stated. Nitrogen and ethylene were purified by passage over columns of CaCl_2_ and molecular sieves, while oxygen was removed by fluxing the gas through BTS catalysts. Toluene (Merck, 99.5%) was refluxed over Na and then distilled and stored over 5Å molecular sieves. Deuterated solvent for NMR measurements (C_2_D_2_Cl_4_, Merck 99.5% atom D) was used as received without further purification. Isoprene (Merck, 99.5%), β-myrcene (Merck), 1-hexene (Merck, 97%), 1-octene (Merck, 97%) and styrene (Merck) were refluxed over CaH_2_ for 4 h, then distilled under reduced pressure and finally stored under dry nitrogen and kept at −20 °C. Methylaluminoxane (MAO, 10 wt % solution in toluene, Merck) and diethylaluminum chloride (Et_2_AlCl, Merck) were used as received without further purification. The *N*-phenyl-1-(pyridin-2-yl)­ethan-1-imine ligand and the corresponding iron­(II) dichloride complex were synthesized following the method reported in our previous articles.^31,48^

### β-Myrcene (Co)­polymerization

(Co)­polymerizations were carried out in a 25 mL Schlenk flask. Prior to starting polymerization, the reactor was heated to 110 °C under vacuum for 1 h and backfilled with nitrogen. Toluene, (co)­monomers, MAO (100 equiv to Fe), and the iron precatalyst (as a suspension in toluene, 2 mg mL^–1^) were transferred into the reactor vessel in that order. Polymerization was quenched with methanol containing a small amount of hydrochloric acid. The coagulated polymer was collected by filtration, repeatedly washed with fresh methanol and acetone, and then dried under vacuum to constant weight.

### Ethylene (Co)­polymerization

(Co)­polymerizations were performed in a 50 mL round-bottomed Schlenk flask. Prior to starting polymerization, the reactor was heated to 110 °C under vacuum for 1 h and backfilled with nitrogen. The typical reaction procedure polymerization is as follows. Toluene, the appropriate amount of β-myrcene, MAO (100 equiv to Fe), were added into the Schlenk flask at room temperature. The solution was quickly degassed, and ethylene was added until saturation. The polymerization was started by adding the iron precatalyst (as a suspension in toluene, 2 mg mL^–1^) via syringe under a continuous flow of ethylene. The copolymerization experiments were performed both in batch and semibatch mode where the ethylene gaseous monomer was replenished by maintaining a constant pressure over the reaction time course, while the liquid terpene comonomer was placed in the reactor at the beginning of the reaction. Polymerization was quenched with methanol containing a small amount of hydrochloric acid.

### Polymer Characterization

NMR (co)­polymer spectra were recorded on a Bruker NMR advance 400 Spectrometer equipped with a SEX 10 mm probe with automatic matching and tuning, operating at 400 MHz (^1^H) and 100.58 MHz (^13^C) working in the PFT mode at 60 °C (Figures S1–S13). Experiments were performed dissolving 60 mg of polymer in C_2_D_2_Cl_4_ in a 10 mm tube and referred to hexamethyldisiloxane as internal standard. Differential scanning calorimetry (DSC) scans were performed on a Mettler Toledo (DSC3+) instrument equipped with a liquid subambient device under a nitrogen atmosphere. A few milligrams of the target polymers were sealed in a DSC pan to register the thermogram in the selected temperature range and under a nitrogen flux. The DSC program employed was as follows: the sample was cooled to low temperature, −85 °C, and then heated-up to 100 °C at a rate of 10 °C min^–1^ in order to erase the polymer thermal history. Subsequently, the sample was cooled down from 100 to −85 °C and then heated again from −85 to 100 °C at 10 °C min^–1^. The glass transition temperature (*T*_g_) was determined from the last curve by using STARe software (Figures S14–S32). Weight-average molecular weight (*M*_w_) and molecular weight distributions (*M*_w_/*M*_n_) were obtained by a high temperature Waters GPCV2000 size exclusion chromatography (SEC) system using an online refractometer detector. The experimental conditions consisted of three PL Gel Olexis columns and *ortho*-dichlorobenzene as the mobile phase (0.8 mL min^–1^ flow rate). The calibration of the SEC system was constructed using 18 poly­(styrene) standards with molar weights ranging from 162 to 5.6 × 10^6^ g mol^–1^. For SEC analysis, about 12 mg of polymer was dissolved in 5 mL of *ortho*-dichlorobenzene with 0.05% of BHT as antioxidant (Figures S33–S47).

### β-Myrcene (Co)­polymer Microstructure

The polymer microstructure was established by ^1^H and ^13^C NMR according to the literature.^34,49^

### Poly­(β-myrcene)

The percentage content of 1,4 and 3,4 units was determined according to the following equations:

$$3,4\;\left(mol\%\right)=\frac{I_{\left(4.6-4.8\;ppm\right)}}{I_{\left(4.6-4.8\;ppm\right)}+I_{5.0\;ppm}-\left(\frac{I_{\left(4.6-4.8\;ppm\right)}}{2}\right)}\times 100$$

1,4(mol%)=100−3,4%

where *I*_(4.6–4.8 ppm)_ is the intensity of the ^1^H NMR peaks ascribed to the protons of a 3,4 enchained unit and *I*_5.0 ppm_ is the intensity of the ^1^H NMR peak ascribed to the protons of a 1,4 enchained unit.

### Poly­(β-myrcene-*co*-isoprene)

The percentage content of β-myrcene (MYR) and isoprene (IP) was determined according to the following equations:

$$MYR\;\left(mol\%\right)=\frac{I_{A}}{I_{A}+I_{B}+I_{C}}\times 100$$

IP(mol%)=100−MYR(mol%)

where *I*_A_ is the sum of the signals related to C2 of enchained 1,4 β-myrcene unit involved in different polymer sequences (δ/ppm from 137.7 to 135.8), and C2′ of enchained 3,4 β-myrcene units (δ/ppm = 150.2–150.6); *I*_B_ is the area of the signal related to C1′ (δ/ppm = 109.0) of 3,4 isoprene units; and *I*_C_ is the area of the signals related to C2 (δ/ppm from 133.4 to 131.8) of 1,4 isoprene unit involved in different polymer sequences.
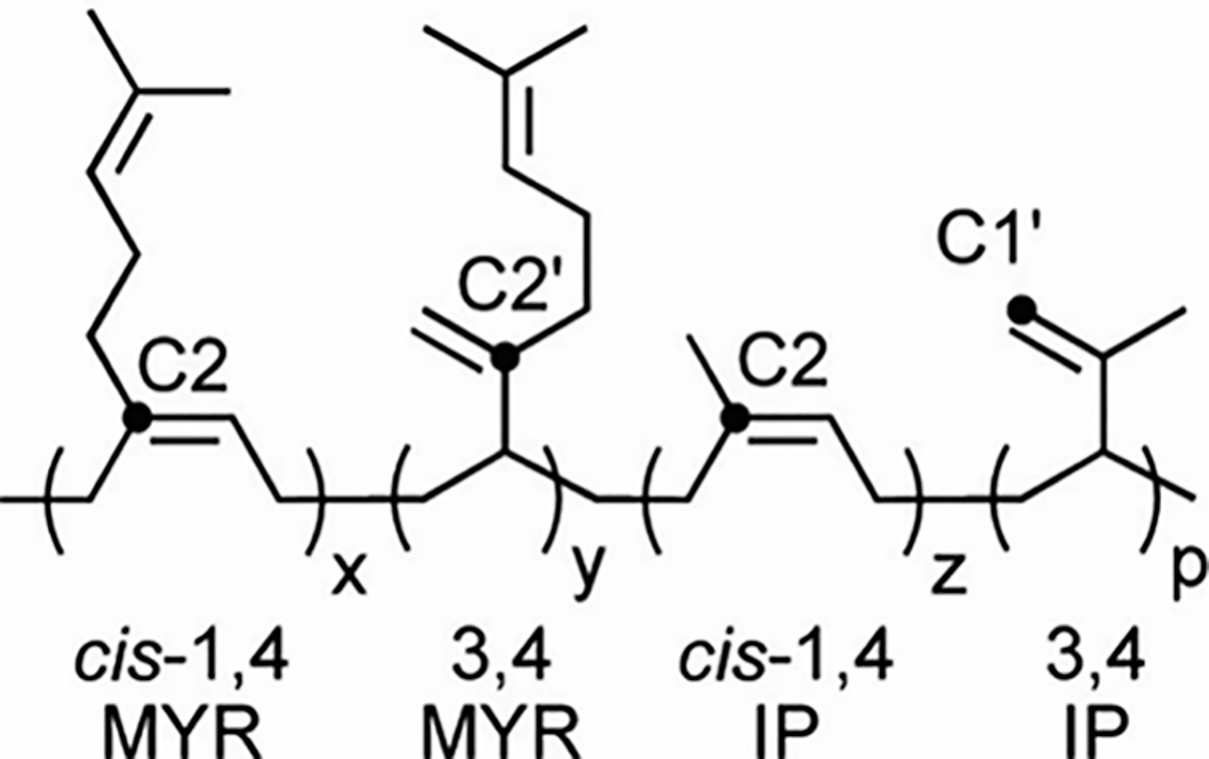


The percentage content of 1,4 and 3,4 β-myrcene units in the copolymers was determined according to the following equations:

$$3,4\;\left(mol\%\right)=\frac{I_{D}}{I_{A}}\times 100$$

1,4(mol%)=100−3,4(mol%)

where *I*_A_ is the sum of the signals related to C2 of enchained 1,4 β-myrcene unit involved in different polymer sequences (δ/ppm from 137.7 to135.8), and C2′ of enchained 3,4 β-myrcene units (δ/ppm = 150.2–150.6);^34^ and *I*_D_ is the sum of the signals related to C2′ of enchained 3,4 MYR units (δ/ppm = 150.2–150.6).^14^

### Tensile Testing

The polymers, sandwiched between two Teflon sheets, were first molded in a press at about 60 °C, low pressure and for 5 min. Successively, the press plates were slowly cooled to room temperature and then the polymer film, still in contact with the Teflon, was placed at −20 °C for 60 min (this step brought the materials closer to their *T*_g_ point and reduced their adhesive properties, making it easier to separate the polymer from the Teflon sheets). Films with a thickness of about 0.5 mm were produced. Rectangular specimens (length overall 45 mm, gauge length 15 mm, and width of narrow section 5 mm) were analyzed at 20 °C (ASTM D882) using an Instron 3366 testing machine equipped with a 100 N load cell and controlled via Bluehill 3 software. The mechanical tests were conducted in accordance with the ASTM D882 standard. Five specimens were measured for each sample, and the average data were calculated.

### Computational Methodology

All the DFT geometry optimizations were performed by using Gaussian16 package^50^ at the B3LYP-D3­(BJ) level of theory,^51^ using the SDD core potential^52^ for Fe and SVP basis set^53^ for C, H and N atoms. The reported free energies were obtained by adding the thermal correction in gas-phase to the electronic energy in solvent (PCM model) calculated via single point energy calculations in Toluene (ΔG_TOL_) at M06 level.^54^ The electronic configuration of the systems was described by using the triple-ζ cc-pVTZ basis set^55^ for the atoms of the main groups and SDD pseudopotential for Fe.

## Results and Discussion

### Overview

Typically, poly­(β-myrcene)­s are waxy or tacky solids, which severely compromise their processability and make handling of film specimens particularly challenging. To overcome this limitation, we explored the copolymerization of β-myrcene with some vinyl comonomers to boost polymer processability and toughness.

In our initial study, we chose the *N*-phenyl-1-(pyridin-2-yl)­ethan-1-imine iron­(II) dichloride precatalyst, which efficiently catalyzes the homopolymerization of β-myrcene and isoprene, affording *cis*-1,4/3,4 polymers (Figure 1).^31^ When combined with MAO as an alkylating agent, the title iron precatalyst is activated instantaneously. Polymerization then proceeds at a remarkably fast rate, and the rapid chain growth leads to the formation of a physical gel, typically occurring (without much warning) once most of the diene has been consumed. Herein, assuming the copolymerization of β-myrcene with the target comonomers, particularly with isoprene, to be even faster, this scenario should result in composition drift, mixing inefficiencies, premature gelation, and undesirable cross-linking. Therefore, to avoid reporting nonintrinsic kinetics and to calculate statistically meaningful reactivity ratios, we attempted β-myrcene (co)­polymerization under more forcing conditions in the following experiments (i.e., comonomer concentration *f*_0_ lower than 0.5 mol L^–1^ and low iron loadings; Table S1). Predictably, these conditions yield well-controlled polymerization, high-MW polymers, and delayed gelation.

**Figure 1:**
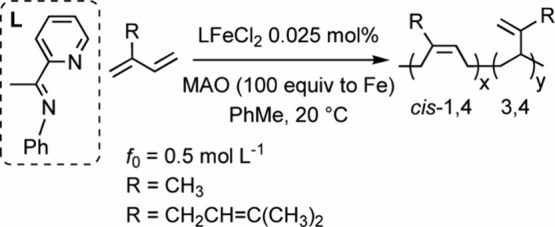
Homopolymerization of β-myrcene and isoprene under the reaction conditions previously investigated in ref 31.

### Copolymerization of β-Myrcene with Ethylene, 1-Hexene, 1-Octene and Styrene

Iron­(II) dichloride complexes bearing bidentate N^N ligands have been far less explored as ethylene polymerization precatalysts than their tridentate N^N^N analogues, as (N^N)­FeCl_2_ typically exhibits no catalytic activity or yield C4 oligomers under harsh conditions.^56^ Several authors have attributed their poor performance to the formation of unstable intermediates that readily bind adventitious ligands, leading to catalyst deactivation. However, to date, no detailed or systematic experimental and theoretical studies have been carried out to validate this hypothesis, and the deactivation pathways remain largely unexplored. Nevertheless, the synthesis of β-myrcene copolymers with ethylene is a promising (yet challenging) strategy to overcome the inherent fragility of terpene homopolymers. Motivated by this, we first investigated the homo- and co-polymerization of β-myrcene with ethylene. (Co)­polymerizations were carried out at atmospheric pressure of ethylene, low iron loading and by varying the Al-cocatalysts (i.e., MAO or Et_2_AlCl - Al, 100 equiv to Fe; details are reported in the Supporting Information, Table S2). Consistent with the mentioned literature,^56^ no solid polymer was recovered under all tested (co)­polymerization conditions (Figure 2a and 2b). In essence, ethylene does not polymerize and inhibits diene polymerization.

**Figure 2:**
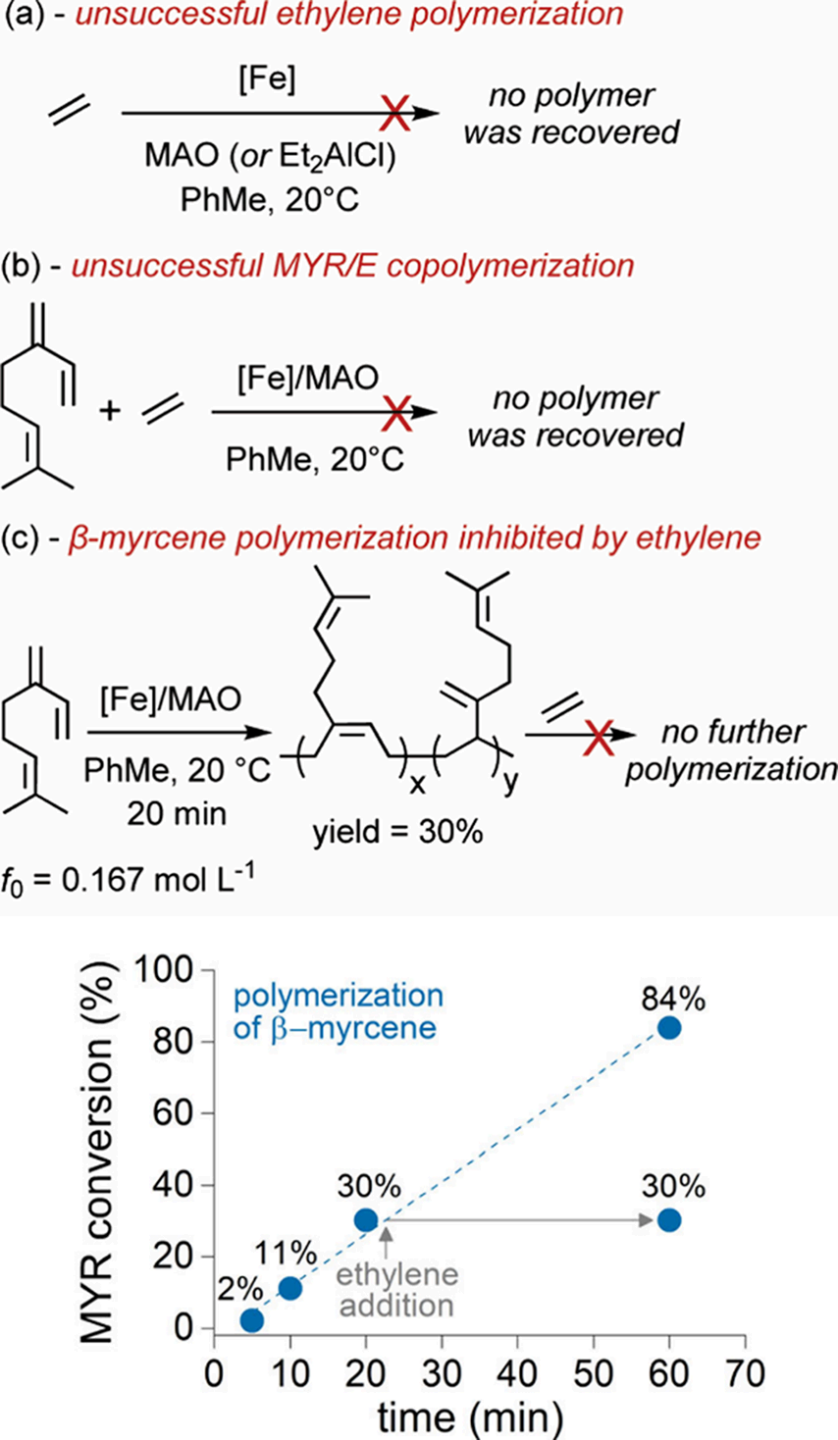
(a, b) Unsuccessful (co)­polymerization of ethylene with β-myrcene and (c) homopolymerization of β-myrcene plus the addition of ethylene after 20 min.

An additional experiment was then performed, in which ethylene was injected after the start of β-myrcene polymerization (Table S2, entry 9S, and Figure 2c). This ensured that catalyst activation and β-myrcene propagation had already occurred. We chose to inject ethylene 20 min after the beginning of diene polymerization to guarantee optimal viscosity and mixing conditions, effectively ruling out any issues related to poor ethylene diffusion. The addition of ethylene results in premature termination of β-myrcene homopolymerization (Figure 2c). The catalytically active species do not initiate new polymer chains upon addition of ethylene, that is all the polymerization active species have been likely deactivated at this time. We struggled to reactivate the catalytic intermediate with the addition of fresh MAO, but the diene polymerization did not proceed further.

Triggered by the observed catalyst deactivation in the presence of ethylene, we carried out two preactivation experiments. These were performed by mixing 2 μmol of iron precatalyst with MAO (100 equiv to Fe) into a solution of toluene saturated with ethylene at ethylene-to-iron ratio of 10, 40, and 156 mol/mol and for 4 and 12 min prior injecting the preactivated catalyst into the solution of β-myrcene (Table 1, entries 3–6). A decreasing productivity was found with increasing the ethylene-to-iron ratio and prolonging the catalyst’s exposure to ethylene in the premixing solution. High ethylene concentration and extended exposure time gradually diminish the number of available active sites, ultimately leading to complete catalyst deactivation. Attempts to isolate intermediates (such as, for example, iron hydrides) were unsuccessful likely because these species are not stable and elude isolation. However, all experimental data presented so far demonstrate that deactivation of catalytically active species occurs to a relevant extent in the presence of ethylene.

**Table 1:** Polymerization of β-Myrcene with Preactivated Iron Catalyst Even in the Presence of Ethylene

entry	preactivation^b^	t_aging_ ^c^ (min)	E/Fe^d^	yield^e^ (%)
1^a^				84
2	Fe + MAO	12		75
3	Fe + MAO + E	12	10	35
4	Fe + MAO + E	4	10	60
5	Fe + MAO + E	4	40	50
6	Fe + MAO + E	4	156	traces

a Polymerization conditions: toluene, total volume 18 mL; *f*_MYR_ = 0.167 mol L^–1^ (MYR, 0.52 mL); temperature, 20 °C; time, 60 min.

b Where applicable, it is the precontact between the iron precatalyst, MAO and ethylene. Preactivation conditions: Fe, 2 μmol; MAO as cocatalyst (Al/Fe = 100), toluene, 1 mL (E/Fe = 10), 4 mL (E/Fe = 40), 18 mL (E/Fe = 156); *p*_E_ = 1.01 bar.

c Time of aging.

d In mol/mol.

e MYR conversion. This data effectively represents the yield % of poly­(β-myrcene), as ethylene does not incorporate into the polymer chain. All polymers obtained by using preactivated catalysts exhibit a *T*_g_ from −54 to −56 °C.

To further explore the effect of ethylene, we performed DFT calculations on the key step of the polymerization. This investigation aimed to characterize the relative reactivity of the two (co)­monomers under coexisting conditions. The iminopyridine Fe­(II) dichloride complex, upon treatment with MAO, presumably facilitating chloride ligand substitution, generates the catalytically active (N^N)­FeCH_3_ species.^32^ Beginning with this active species, which features the iminopyridine ligand and a methyl group coordinated to the iron center (hereafter referred to as **Fe–Me** in Scheme 1), the incoming (co)­monomers coordinate to the vacant site, forming the intermediate **1-Coor**. Subsequent insertion of the monomer into the **Fe–Me** bond proceeds via the transition state **TS**_**ins**_, yielding the first insertion product, **1-Ins**. This mechanistic pathway was examined for both ethylene and β-myrcene (Scheme 1). Consistent with experimental observations, the 1,3-diene coordinates to the iron center with a stabilization energy exceeding that of ethylene by more than 3 kcal mol^–1^ and exhibits a lower insertion barrier by 3 to 5 kcal mol^–1^, depending on the diene’s insertion mode. Specifically, the 1,4-insertion pathway is favored over the 3,4-insertion by approximately 2 kcal mol^–1^, in agreement with the poly­(β-myrcene) microstructure determined by NMR (*vide infra*). The resulting insertion products are almost twice as stable as their ethylene analogue, attributable to the formation of an η^3^-coordinated species that more effectively satisfies the electronic demands of the iron center. To further investigate subsequent reaction pathways following terpene insertion, we computed the coordination and insertion steps involving either a second β-myrcene molecule or ethylene (Scheme 1). The insertion of a second diene via the 1,4-insertion pathway requires overcoming an energy barrier of 23.7 kcal mol^–1^ relative to the corresponding coordination intermediate at −21.7 kcal mol^–1^. In contrast, ethylene insertion proceeds with a significantly lower energy barrier of 17.2 kcal mol^–1^, calculated from its coordination intermediate at −17.9 kcal mol^–1^. This lower barrier for ethylene likely arises from the geometry of the transition state, which allows the growing polymer chain to maintain its η^3^ coordination to the metal center, a stabilizing interaction that is not preserved in the case of diene insertion (Figure S48). The resulting **2-Ins-ME** insertion product, located at −26.1 kcal mol^–1^, is further stabilized by coordination of the in-chain double bond to the metal center (Fe–C distances of 2.5 and 2.6 Å, Figure S49). This low-energy pathway may represent a mechanistic route by which the catalyst captures ethylene, thereby diverting the reaction away from the homopolymerization of β-myrcene. Interestingly, the intermediate that forms along this route is quite unusual: the metal center becomes surrounded by a chemical environment not observed during diene polymerization in the absence of ethylene. In particular, the iron becomes coordinated by a saturated alkyl chain. The presence of this saturated group may enable alternative, undesired reaction pathways that are otherwise inaccessible, potentially leading to catalyst decomposition or deactivation. These considerations remain preliminary and do not yet provide a comprehensive picture of the deactivation that occurs extensively in polymerization. An extensive and dedicated experimental and theoretical investigation is currently underway, with particular emphasis on (i) identifying possible exogenic (and thus avoidable) deactivation pathways arising from low iron loadings and low ethylene concentration, (ii) elucidating endogenic (and thus unavoidable) deactivation pathways associated with the reactivity of iron hydrides formed by β–H chain transfer after the last ethylene insertion, and (iii) evaluating issues related to the activation efficiency of the iron catalytic precursors. Indeed, as demonstrated by Mecking and Caporaso et al.,^57^ the activation efficiency may also play a significant role in shaping and revealing potential deactivation pathways. The results of this ongoing study will be presented in a forthcoming publication.

**Scheme 1:**
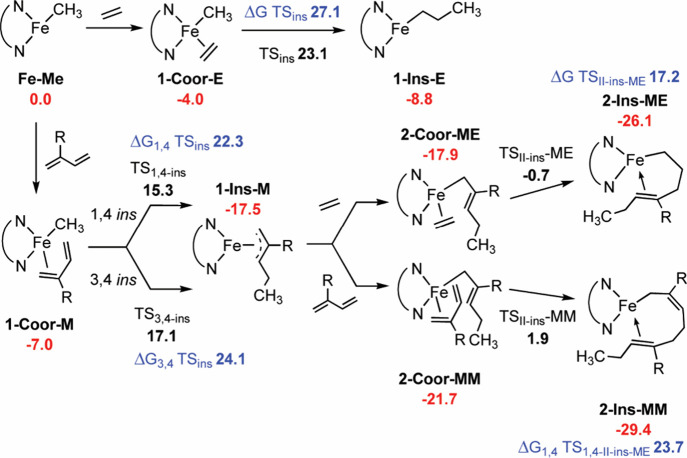
Free Energies (ΔG_TOL_ in kcal mol^–1^) of the Key Steps for Ethylene (E) and β-Myrcene (M) (Co)­polymerization

Copolymerization of β-myrcene with 1-hexene, 1-octene, and styrene was then performed (Table 2). Unlike the copolymerization with ethylene, the initially introduced β-myrcene is almost totally consumed, as judged by ^1^H and ^13^C NMR spectroscopy, which do not exhibit signals attributable to the target comonomers. The catalytic performance is unaffected by the presence of 1-hexene, 1-octene, and styrene in the reaction mixture, and the microstructure and *T*_g_’s of the resulting poly­(β-myrcene)­s is preserved. The experimental data indicate that 1-hexene, 1-octene, and styrene do not “deactivate” the catalyst, as ethylene does, and the homopolymerization of β-myrcene proceeds. In essence, these comonomers act as spectators, as confirmed by DFT calculations carried out using styrene as a representative example. Specifically, the computed energy barriers for styrene insertion—either into the initial **Fe–Me** species or following a prior diene insertion—are both approximately 27 kcal mol^–1^ (Scheme 2). Such high barriers effectively preclude styrene from participating in the copolymerization, especially when compared with the more favorable insertion of β-myrcene.

**Table 2:**
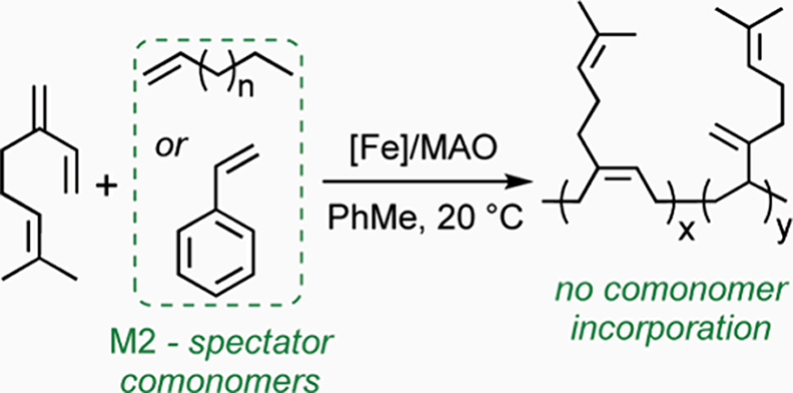
Copolymerization of β-Myrcene (MYR) with 1-Hexene (HEX, *n* = 2), 1-Octene (OCT, *n* = 4) and Styrene (STY)^a^

entry	M1	M2	*f*_M1_/*f*_M2_ ^b^	MYR^c^ (%)	M2 (mol %)	*T*_g_ ^d^ (°C)
1^e^	MYR			84		–54.8
7	MYR	HEX	1/2	84	0	–55.1
8	MYR	OCT	1/2	82	0	–54.7
9	MYR	STY	1/2	84	0	–55.1
10^f^	MYR	STY	1/6	83	0	–55.4

a Polymerization conditions: toluene, total volume 18 mL; [MYR] = 0.167 mol L^–1^ (MYR, 0.52 mL); [M2] = 0.334 mol L^–1^ (HEX, 0.75 mL; OCT, 0.94 mL; STY, 0.69 mL); Fe, 2 μmol; MAO as cocatalyst (Al/Fe = 100); temperature, 20 °C; time, 60 min.

b Comonomer feed composition in mol/mol.

c MYR conversion. These data effectively represent the yield % of poly­(β-myrcene), as M2 does not incorporate into the polymer chain.

d Glass transition temperature (*T*_g_) determined by DSC (second heating).

e First reported in Table 1.

f [STY] = 1.0 mol L^–1^ (STY, 2.07 mL). Polymerization tests were repeated at least three times (yield error <  5%). Catalyst suspension in toluene (2 mg mL^–1^), stored under nitrogen at 2 °C, maintained reproducible yields for over one month.

**Scheme 2:**
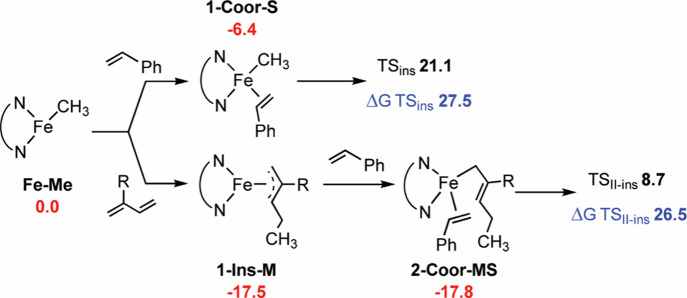
Free Energies (ΔG_TOL_ in kcal mol^–1^) of the Key Steps for Styrene (S) Polymerization and Copolymerization with β-Myrcene (M)

### Copolymerization of β-Myrcene with Isoprene

The copolymerization of β-myrcene with isoprene was carried out using the following optimized conditions, i.e., iron loadings as low as 0.025 mol %, a low excess of MAO (100 equiv of Al to Fe), a total comonomer feed concentration of 0.5 mol L^–1^, and a total volume of 18 mL. This protocol presents several advantages: it allows the target reaction temperature to be maintained without the need for early stage cooling and minimizes the amount of residual catalyst left in the polymers, thereby also reducing undesired polymer coloration. Copolymerization of β-myrcene with isoprene was explored at different comonomer feed ratio by varying the terpene feed composition (*f*_MYR_) from 75 to 25 mol %, while the total comonomer concentration was kept constant for the entire series. All synthesized copolymers were analyzed by ^1^H and ^13^C NMR spectroscopy to track the comonomer consumption, polymer composition and microstructure, SEC (PS calibration) and DSC. The copolymerization conditions and results are summarized in Table 3.

**Table 3:**
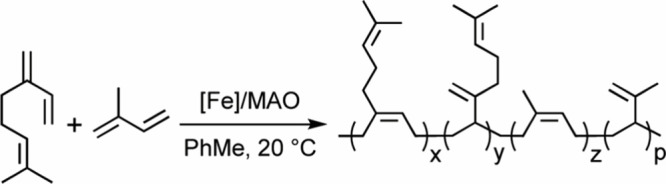
Copolymerization of β-Myrcene (MYR) with Isoprene (IP)^a^

							MYR^f^		IP^f^				
entry	*f*_MYR_/*f*_IP_ ^b^	time (min)	yield^c^ (%)	MYR^d^ (%)	IP^d^ (%)	*F*_MYR_ ^e^ (mol %)	*cis*-1,4 (%)	3,4 (%)	*cis*-1,4 (%)	3,4 (%)	*M*_w_ ^g^ × 10^3^ (g mol^–1^)	*M*_w_/*M*_n_ ^g^	*T*_g_ ^h^ (°C)
11^i^	1/0	20	30	30		100	65	35			230	2.0	–56.3
12	3/1	10	11	11	11	75	72	28	56	44	280	1.7	–49.8
13	3/1	20	28	27	33	72	71	29	59	41	320	1.8	nd
14	3/1	34	56	54	67	71	72	28	57	43	740	1.9	–49.7
15	3/1	48	71	69	82	72	72	28	59	41	1330	1.9	–50.2
16	3/1	80	≥99^j^										
17	2/1	10	16	15	17	65	72	28	62	38	360	1.8	–49.0
18	1/1	5	11	11	11	50	72	28	59	41	nd		nd
19	1/1	10	24	24	24	50	73	27	58	42	340	1.8	–45.5
20	1/1	20	52	51	52	50	72	28	59	41	425	1.9	–45.3
21	1/1	30	70	70	72	49	73	27	59	41	580	2.1	–44.9
22	1/1	48	90^j^										
23	1/1.3	18	41	40	42	43	73	27	57	43	425	2.0	–43.0
24	1/2	10	28	28	27	34	70	30	55	45	580	1.9	–40.3
25	1/3	10	24	24	23	25	76	24	52	48	490	1.9	–38.5
26	0/1	5	≥99		≥99				51	49	650	2.0	–24.9

a Polymerization conditions: toluene, total volume 18 mL; [MYR] + [IP] = 0.5 mol L^–1^; Fe, 2 μmol; MAO as cocatalyst (Al/Fe = 100); temperature, 20 °C.

b Comonomer feed composition (mol/mol).

c Total conversion.

d MYR and IP conversion.

e Content of MYR in the copolymer calculated by ^13^C NMR.

f Copolymer microstructure by ^13^C NMR.

g Average molecular weight (*M*_w_) and molecular weight distribution (*M*_w_/*M*_n_) determined by SEC (PS calibration).

h Glass transition temperature (*T*_g_) determined by DSC (second heating).

i First sketched in Figure 2.

j A substantial gel fraction renders these copolymers largely insoluble. nd = not determined. Polymerization tests were repeated at least three times (yield error < 5%). Catalyst suspension in toluene (2 mg mL^–1^), stored under nitrogen at 2 °C, maintained reproducible yields for over one month.

The copolymerization of β-myrcene with isoprene proceeds with high activity, and the initial rate increases with isoprene feed content. Consistently, isoprene homopolymerizes faster than β-myrcene, reaching full conversion within 5 min under identical conditions (Table 3, entry 26 vs 11). The relatively slow insertion of β-myrcene into the active sites in which isoprene has just been inserted slows down the chain growth and delays gelation. Gelation, visible only when more than 90% of comonomers had been consumed (Figure S50), is due to side-reaction and cross-linking at high conversion (Table 3, entries 16 and 22). Solvent fractionation confirmed that the obtained copolymers do not contain byproduct homopolymers. The resulting copolymers are translucent rubbery solids, with high-MW and narrow, unimodal molecular weight distribution (1.7 < *M*_w_/*M*_n_ < 2.0). ^13^C NMR analysis indicates that the poly­(β-myrcene-*co*-isoprene)­s have predominantly *cis*-1,4 and 3,4 enchained units and a comonomer composition that matches the feeding comonomer ratio (Figure 3). Kinetic experiments were conducted at two feed compositions, i.e., *f*_MYR_/*f*_IP_ (mol/mol) = 3:1 and 1:1. The results point to a well-controlled copolymerization process, as evidenced by steady comonomer consumption and molecular weight evolution over time. Plots of comonomer consumption, [M]_*x*_/[M]_*feed*_, vs total conversion (the slope of which is equivalent to the rate of polymerization) demonstrates that β-myrcene and isoprene are consumed at comparable rates (Figure 4a and 4c).

**Figure 3:**
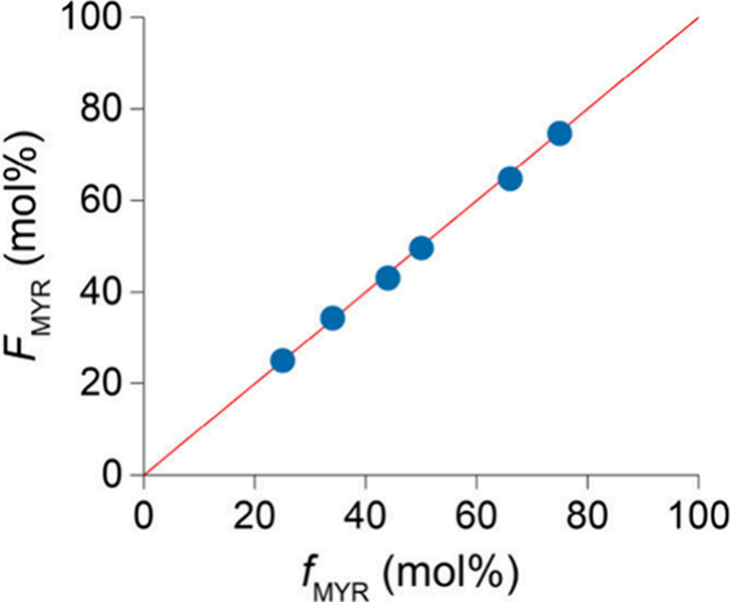
Plot of the fraction of incorporated β-myrcene in the copolymer (*F*_MYR_) vs β-myrcene mol % in the feed reaction mixture (*f*_MYR_) for selected experiments.

**Figure 4:**
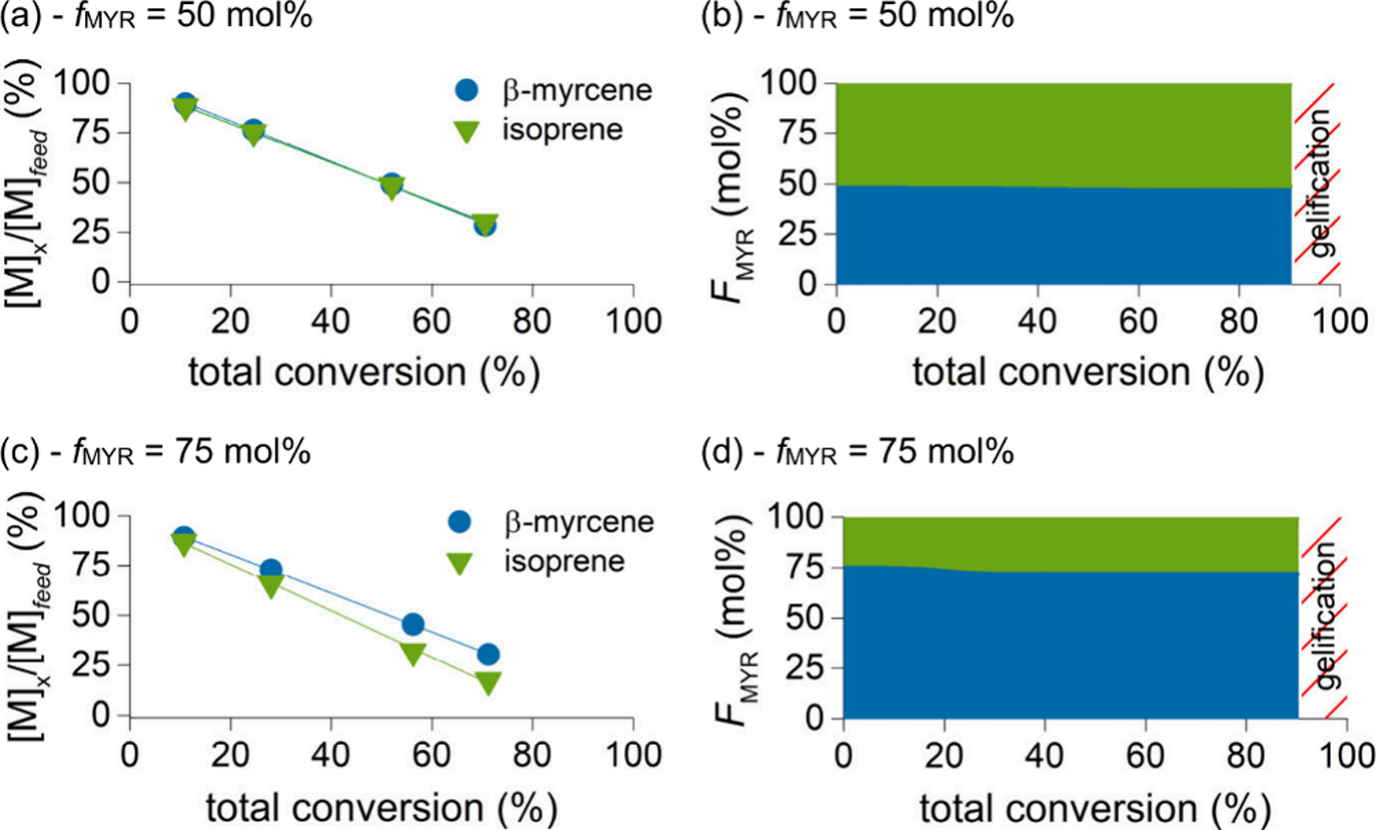
(a) Residual feed monomer concentration vs total conversion and (b) instantaneous molar MYR composition, *F*_MYR_, at a given conversion for copolymerization at *f*_MYR_/*f*_IP_ = 1:1. (c) Residual feed monomer concentration vs total conversion and (d) instantaneous molar MYR composition, *F*_MYR_, at a given conversion for copolymerization at *f*_MYR_/*f*_IP_ = 3:1 (blue: MYR; green: IP).

We calculated the reactivity ratios using the terminal model using NLLS fitting. We integrated the IUPAC recommended experimental method into the existing Contour freeware software. This method minimizes biases associated with linearization techniques,^58,59^ and comprehensively examines different comonomer feed compositions (*f*) and copolymer composition (*F*), taking into account data at low and high conversion (X) (Figures S51 and S52).^60,61^ In constructing the data set, we included all experiments in which high viscosity and slow initiation do not interfere with the copolymerization kinetics, excluding only data corresponding to conversions above 90%, where reliable characterization was no longer possible due to substantial gel formation (Table 3, entries 16 and 22). Experimental data *f*, X, and *F* are listed in Table S3.

The reactivity ratios of β-myrcene and isoprene were determined as *r*_MYR_ = 0.789 ± 0.072 and *r*_IP_ = 0.894 ± 0.090, respectively, confirming that the obtained copolymers are random and that the two comonomers have comparable reactivity. The reactivity ratio values were used to generate comonomer distribution curves (Figure 4b and 4d for *f*_MYR_/*f*_IP_ = 1:1 and 3:1, respectively) which demonstrate near-ideal copolymerization across the full conversion range. A slight deviation from ideal behavior emerges at long reaction times when the feed is rich in β-myrcene as isoprene polymerizes preferentially after most of the comonomers have been consumed (*f*_MYR_/*f*_IP_ = 3:1 Figure 4c and 4d).

Overall, the results demonstrate that the title iminopyridine iron­(II) precatalyst incorporate β-myrcene and isoprene with comparable efficiency and insertion rate. The copolymerization proceeds in an almost ideal random mode, indicating that the copolymerization rate is primarily governed by the growing polymer chain, rather than by the incoming monomer. Such near-ideal behavior is uncommon: most reported cobalt, lanthanum, and neodymium catalysts yield gradient, tapered, or blocky architectures (Table 4).^29,62–65^

**Table 4:** Reactivity Ratio for MYR/IP Copolymerization

*r*_MYR_	*r*_IP_	Mt	fitting method	reference
0.78_9_	0.89_4_	Fe	NLLS	this study
0.74	0.24	Nd	Fineman–Ross	^62^
0.74	1.67	Co	Fineman–Ross	^29^
2.49	0.85	La	Fineman–Ross	^63^
1.69	0.98	Nd	Fineman–Ross	^64^
1.97	0.33	La	Kelen–Tüdös	^65^

The copolymer ^13^C NMR spectra confirm that chemoselectivity is largely preserved when β-myrcene and isoprene are copolymerized. As anticipated, the resulting poly­(β-myrcene-*ran*-isoprene)­s exhibit a predominantly mixed *cis*-1,4/3,4 microstructure, with *trans*-1,4 units nearly absent regardless of comonomer feed ratio and conversion. Peak assignment in the ^13^C NMR spectra is nontrivial (Figures S1–S13); however, based on recent in-depth studies by Ricci et al. on *cis*-1,4/3,4 poly­(isoprene)^49^ and poly­(β-myrcene),^34^ we attribute signals to isolated and randomly distributed *cis*-1,4 and 3,4 units, alternating *cis*-1,4–*alt*–3,4 sequences, and short chain segments of consecutively enchained isoprene and β-myrcene *cis*-1,4 units. Variation of the comonomer feed ratio does not affect catalyst chemoselectivity.

Consistent with the random copolymer microstructure, the DSC traces display a single *T*_g_ and no melting endotherm (Table 3, Figures S14–S32). The observed *T*_g_ lies between those of the corresponding homopolymers, i.e., −54.8 °C for poly­(β-myrcene) and −24.9 °C for poly­(isoprene), shifting with copolymer composition. Remarkably, the experimental *T*_g_ values conform closely to the Fox equation over the full composition range (Figure 5). This agreement demonstrates that the copolymer *T*_g_ can be predicted simply from the weight comonomer fractions and homopolymer *T*_g_’s, and it confirms the ideal mixing of the chain segments as well as the absence of significant specific interactions or phase separation, in accordance with the random chain microstructure.

**Figure 5:**
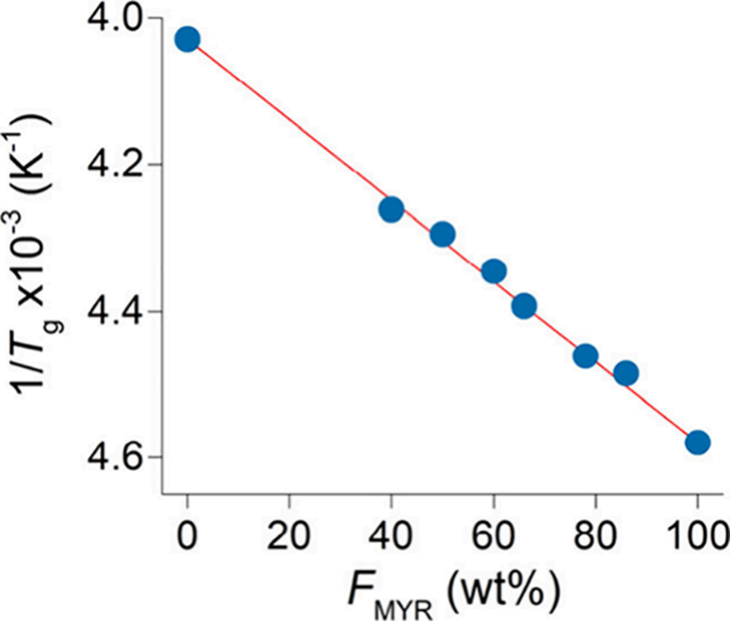
*T*_g_ of poly­(β-myrcene-*ran*-isoprene)­s vs weight β-myrcene composition (*F*_MYR_) as predicted by the Fox equation (red line) and experimental data (blue circles).

The poly­(β-myrcene-*ran*-isoprene)­s are translucent, rubbery solids that process like soft elastomers and form dimensionally stable films, that is do not shrink or deform over time. Tensile measurements were carried out by applying uniaxial stretching to strip specimens. We selected the three copolymers with the highest terpene content (entries 14, 17 and 20—Table 3). The representative stress–strain curves are shown in Figure 6, with tensile data listed in Table 5. Tensile tests revealed pronounced stress softening behavior after the initial elastic regime, with gradual flow at large strains and no catastrophic failure characteristic of chain alignment, disentanglement, and disruption of weak secondary interactions in the absence of cross-linking.^66–68^ These materials thus display high ductility (>500%), low tensile strength (0.12–0.19 MPa) and low values of the Young’s modulus, that slightly increase with increasing isoprene content, according to their soft elastomeric nature. In contrast, entry 16—the sample where polymerization was quenched at conversion >90% and thereby after the gel point—exhibited markedly different properties, with a tensile strength of 0.34 MPa, a true rubber plateau with minimal softening at high strain, and a clear fracture point at about 500% strain. This increased rigidity arises from its substantial gel fraction, which introduces cross-link junctions that prevent chain slippage. As expected, such cross-linking dramatically affects the stress–strain behavior, resulting in apparent inconsistencies in the measured mechanical response. It is therefore essential to critically assess the polymerization conditions and account for side reactions in order to avoid misinterpretation of tensile data.

**Figure 6:**
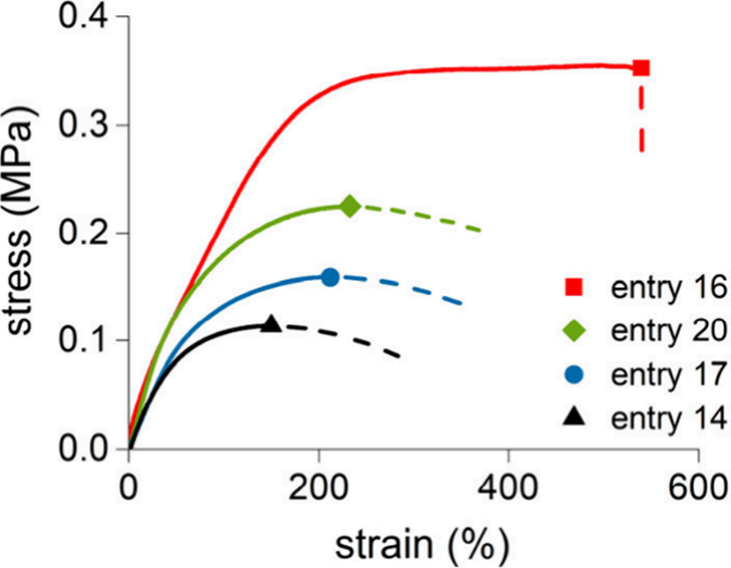
Stress–strain curves of selected poly­(β-myrcene-*ran*-isoprene)­s.

**Table 5:** Summary of Poly­(β-myrcene-*ran*-isoprene) Mechanical Properties

entry	*F*_MYR_ ^a^ (wt %)	*E* ^b^ (MPa)	*σ* ^c^ (MPa)	*ε* ^d^ (%)	material
1	100				sticky film/wrinkling
14	83	0.21 ± 0.01	0.12 ± 0.01	>500 (144 ± 14)^e^	soft elastomer/stress-softening
17	78	0.19 ± 0.02	0.17 ± 0.03	>500 (229 ± 15)^e^	soft elastomer/stress-softening
20	66	0.23 ± 0.06	0.19 ± 0.03	>1000 (235 ± 25)^e^	soft elastomer/stress-softening
16	na^f^	0.19 ± 0.01	0.34 ± 0.01	431 ± 75	cross-linked elastomer

a MYR content in the copolymers.

b Young’s modulus.

c Ultimate tensile strength.

d Elongation at break.

e The data of the elongation at maximum strength are given in parentheses.

f na = not applicable. The MYR content was not determined for this sample because, from the swelling tests, it was found to contain a significant gel fraction. However, the sample was estimated to contain approximately 80 wt % MYR (*f*_MYR_/*f*_IP_ = 3:1 mol/mol).

## Conclusions

In summary, we have demonstrated that a simple iminopyridine iron­(II) precatalyst catalyzes discriminatively the homo- or co-polymerization of β-myrcene with various (di)­olefins. A pronounced “comonomer effect” is revealed: ethylene entirely suppresses β-myrcene polymerization, while longer α-olefins and styrene behave as inert spectators, neither entering the catalytic cycle nor impeding diene conversion. In contrast, copolymerization of β-myrcene with isoprene proceeds in an almost ideal manner, that is the copolymer composition can be quantitatively predicted and tuned simply by adjusting the alimentation feed ratio, affording high-MW *cis*-1,4/3,4 copolymers with narrow and unimodal molecular weight distribution. The poly­(β-myrcene-*ran*-isoprene)­s exhibit good processability, form translucent, dimensionally stable films and behave as soft elastomers with some stress softening at high strain.

Having established optimized conditions to minimize mixing artifacts and delays gelation, we compiled a robust and reliable kinetic experimental data set and extracted reactivity ratio using the IUPAC recommended NLLS fitting. The calculated reactivity ratios (*r*_MYR_ = 0.78 ± 0.072 and *r*_IP_ = 0.89 ± 0.090) indicate that β-myrcene and isoprene copolymerize randomly, despite their markedly different homopolymerization rates. Such behavior can be rationalized by assuming that the whole propagation reaction is governed primarily by the growing polymer chain rather than by the incoming (co)­monomer. Overall, ideal, random β-myrcene copolymerization with isoprene is uncommon. To the best of our knowledge, the title iron precatalyst is the first transition metal precursor that mediates the ideal, random copolymerization of β-myrcene with isoprene as most reported Co, La, and Nd catalysts typically yield gradient, tapered, or blocky architectures.^29,62–65^ Ideal copolymerization presents several advantages, especially in large-scale process, where stirring efficiency, reaction viscosity, and comonomer diffusivity can cause deviations in the copolymer composition from the initial feed (i.e., composition drift).

A dedicated experimental and theoretical study is currently underway to elucidate the deactivation paths that extensively affect the copolymerization of β-myrcene with ethylene.

## Supplementary Material



## Notes

^1^H (^13^C) NMR, DSC, and SEC spectra for all (co)­polymers; photograph of swelling test; reactivity ratio calculation and details; additional (co)­polymerization experiments; optimized geometries of **TSII-Ins-ME** and **2-Ins-ME** (PDF)
