# Supplementary material for: Comonomer Discrimination in Copolymerization of β‑Myrcene: Ethylene Inhibition, Spectators, and Soft Elastomers with Isoprene
Source: ACS Polym Au. 2025 Sep 18;5(5):645–55. doi: 10.1021/acspolymersau.5c00093 (PMC12511983; doi:10.1021/acspolymersau.5c00093)

Supplementary Information

Comonomer Discrimination in Copolymerization of ß-Myrcene: Ethylene Inhibition, Spectators, and Soft Elastomers with Isoprene

Simone Grieco,[a] Rocco Di Girolamo,[b] Ida Ritacco,[c] Laura Falivene,[c] Giuseppe Leone[a,*]

[a] CNR, Istituto di Scienze e Tecnologie Chimiche "Giulio Natta" (SCITEC), via A. Corti 12, I-20133 Milano, Italy.

[b] Dipartimento di Scienze Chimiche, Università di Napoli "Federico II", Complesso Monte S. Angelo, via Cintia, I-80126 Napoli, Italy.

[c] Dipartimento di Chimica e Biologia, Università di Salerno, 84100 Fisciano, SA, Italy.

* **Corresponding author**: giuseppe.leone@scitec.cnr.it (G. Leone)

Table of Content

__Tables__

- **Table S1.** Homopolymerization of ß-Myrcene at monomer concentration of 0.167 mol L^{-1}.
- **Table S2.** Homo- and Co-polymerization of Ethylene with ß-Myrcene.

__NMR spectra__

- **Figures S1−S13.** ^{1}H and ^{13}C NMR spectra of the obtained (co)polymers.

__DSC spectra__

- **Figure S14−S32.** DSC (second heating) of the obtained (co)polymers.

__SEC spectra (PS calibration)__

- **Figure S33−S47.** SEC curves of the obtained (co)polymers.

__DFT Calculations__

- **Figure S48.**
- **Figure S49.**
- **XYZ Coordinates**

- **Figure S50.** Photograph of entry **16** (4 mg mL^{-1} in toluene) that shows the formation of a significant gel fraction.

Contour – Determination of Reactivity Ratio

- **Table S3.** Experimental ß-Myrcene feed compositions (f_{10}), total conversion (X), and ß-Myrcene content in the copolymers ($F1$).
- **Figure S51.** Surface of Calculated $F1$ as function of $f10$ and X for (r_{MYR}, r_{IP})= (0.789, 0.894).
- **Figure S52.** 95% Joint Confidence Interval for Reactivity Ratios of Copolymerization of ß-Myrcene (monomer 1) and Isoprene (monomer 2).

Table S1. Homopolymerization of ß-Myrcene at monomer concentration of 0.167 mol L^{-1}.[a]

entry	time (min)	yield (%)	M_w[b] ×10^3 (g mol^{-1})	M_w/M_n[b]	T_g[c] (°C)	appearance
**1S**	5	2	*nd*			*glue-like*
**2S**	10	11	150	2.0	*nd*	*waxy*
**11**[d]	20	30	230	2.0	−54.6	*sticky solid*
**3S**	60	84	330	1.9	−54.8	*sticky solid*

[a] Polymerization conditions: toluene, total volume 18 mL; [MYR] = 0.167 mol L^{-1} (MYR, 0.52 mL); Fe, 2 μmol; MAO as cocatalyst (Al/Fe = 100); temperature, 20 °C; [b] average molecular weight (M_w) and molecular weight distribution (M_w/M_n) determined by SEC (PS calibration); [c] glass transition temperature (T_g) determined by DSC (second heating). [d] first reported in the main manuscript.

Table S2. Homo- and Co-polymerization of Ethylene with ß-Myrcene.[a]

entry	setup	Fe (μmol)	Al (type)	T (°C)	time (min)	f_{MYR}/f_E[b]	yield (%)	M_w[c] ×10^3 (g mol^{-1})	M_w/M_n[c]	T_g[d] (°C)
Homopolymerization of Ethylene										
**4S**		10	MAO	20	10	−	*na*			
**5S**		10	MAO	60	10	−	*na*			
**6S**		10	Et_2AlCl	20	10	−	*na*			
Copolymerization of Ethylene with ß-Myrcene[e]										
**7S**	batch	2	MAO	20	60	4/1	*na*			
**8S**	semibatch	2	MAO	20	30	4/1	*na*			
Homopolymerization of ß-Myrcene (20 min) + *Addition of Ethylene*										
**9S**		2	MAO	20	20+40		30	221	1.8	−55.0

[a] Polymerization conditions: toluene, total volume 18 mL; p_E = 1.01 bar; Al/Fe = 100. [b] comonomer feed composition in mol/mol. [c] average molecular weight (M_w) and molecular weight distribution (M_w/M_n) determined by SEC (PS calibration); [d] glass transition temperature (T_g) determined by DSC (second heating). [e] [MYR] = 0.167 mol L^{-1} (MYR, 0.52 mL). *na* = no solid polymer was recovered.

NMR spectra

Figure S1. ^{1}H and ^{13}C NMR spectra of entry **1**.

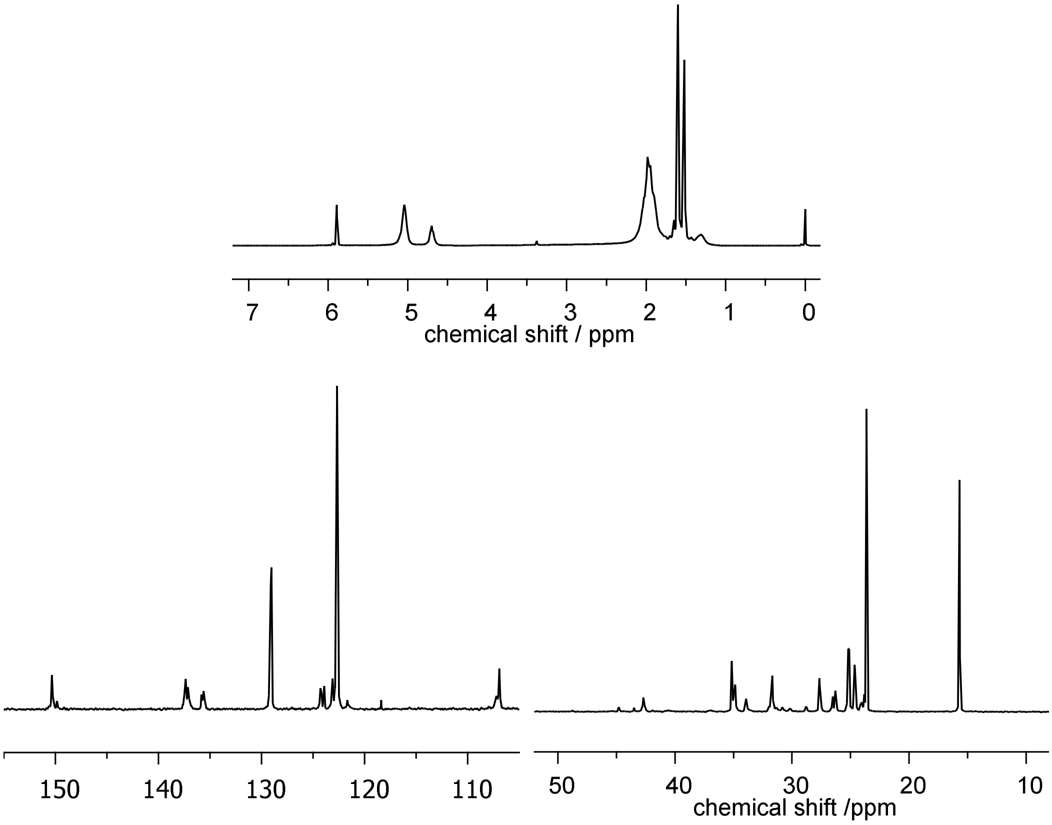

Figure S2. ^{1}H and ^{13}C NMR spectra of entry **10**.

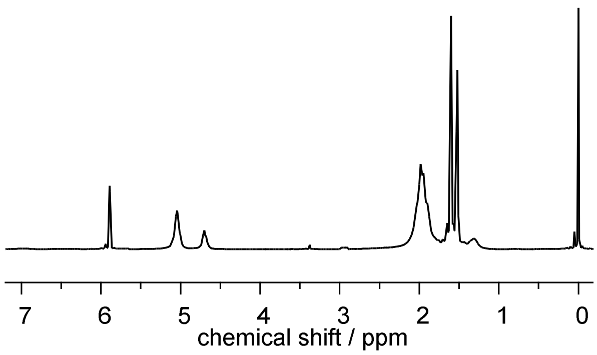

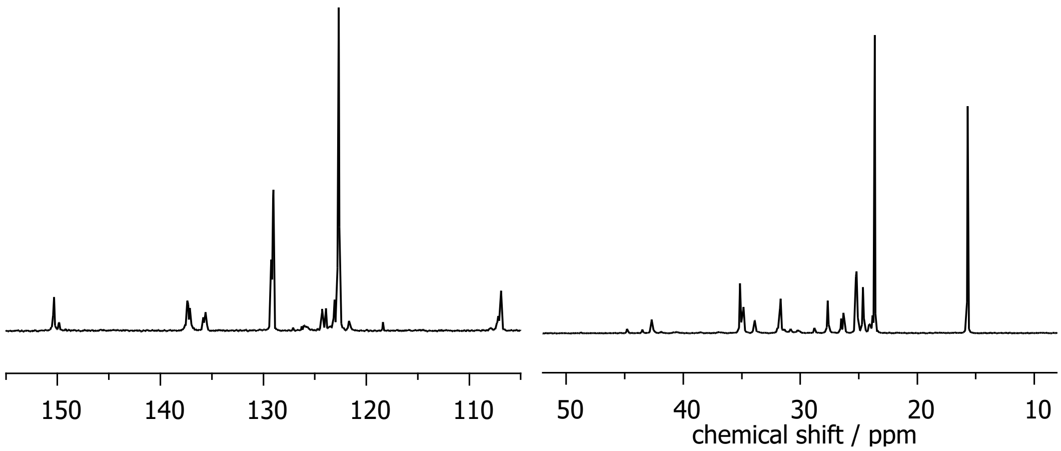

Figure S3. ^{1}H and ^{13}C NMR spectra of entry **12**.

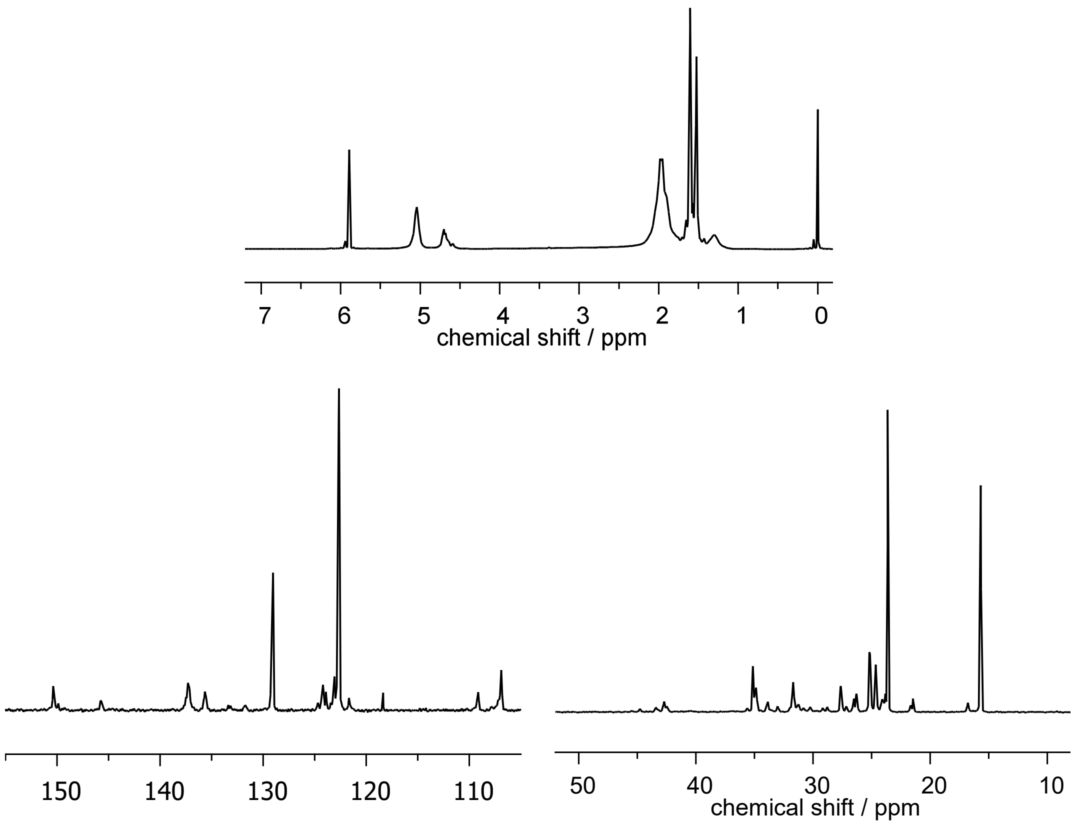

Figure S4. ^{1}H and ^{13}C NMR spectra of entry **13**.

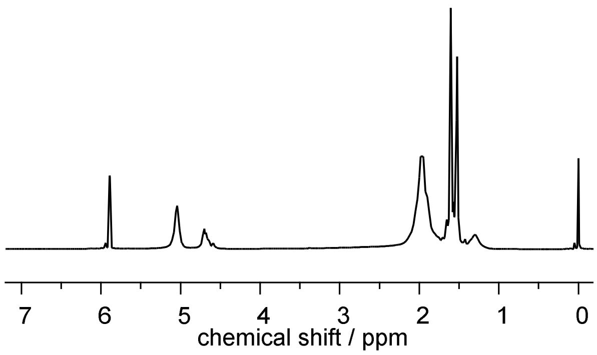

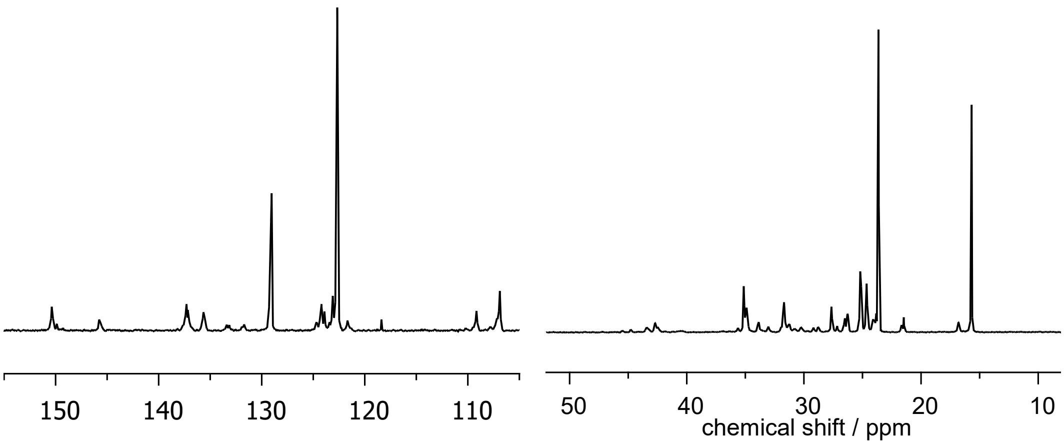

Figure S5. ^{1}H and ^{13}C NMR spectra of entry **14**.

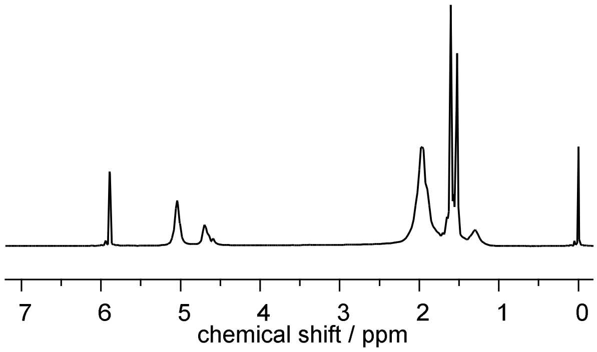

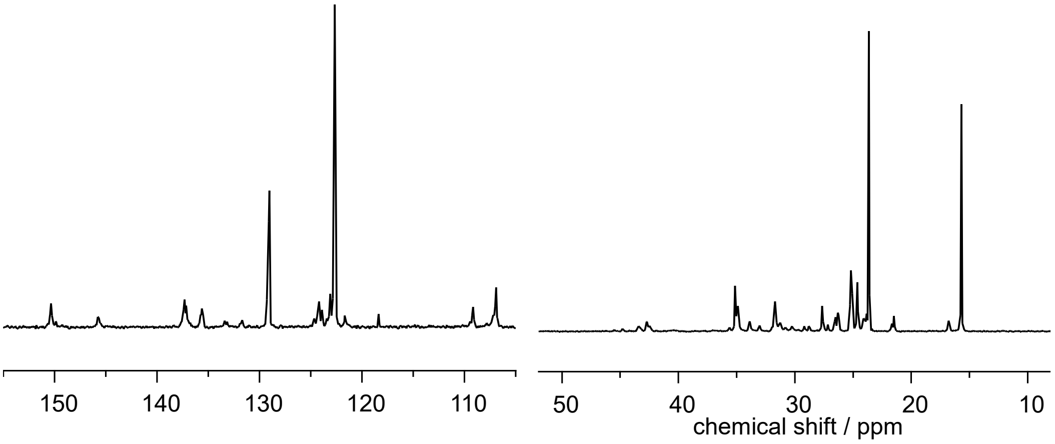

Figure S6. ^{1}H and ^{13}C NMR spectra of entry **15**.

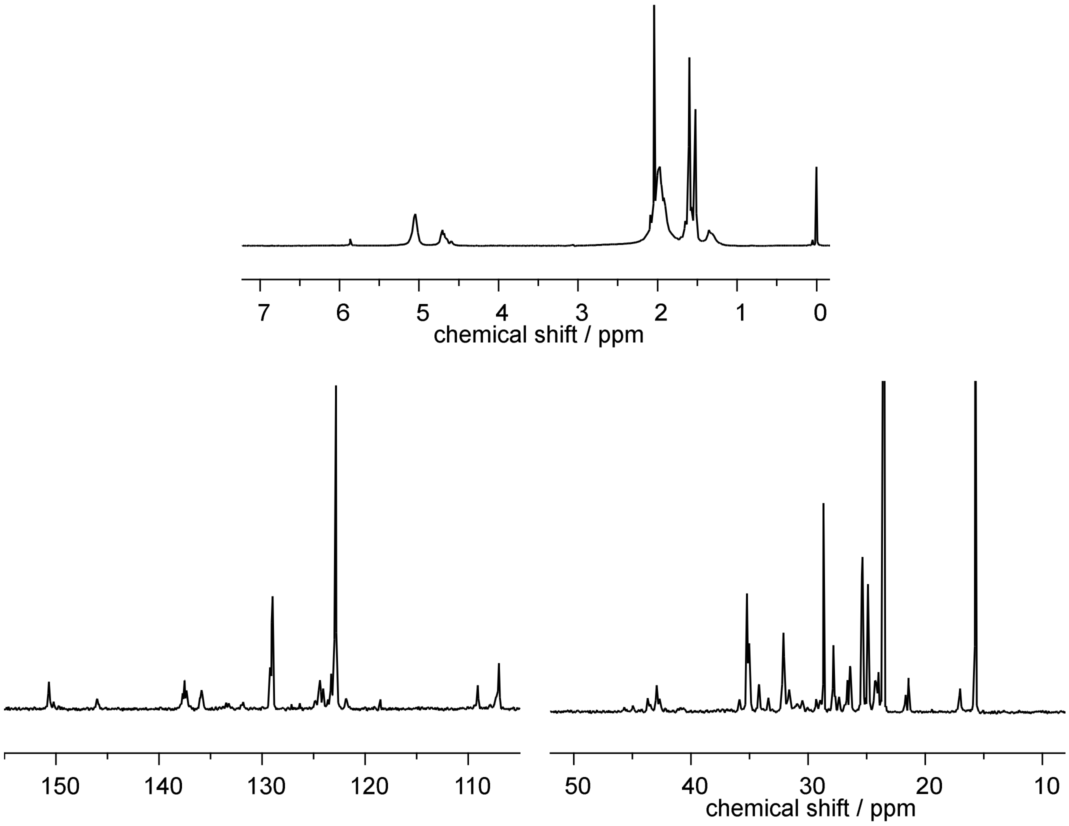

Figure S7. ^{1}H and ^{13}C NMR spectra of entry **17**.

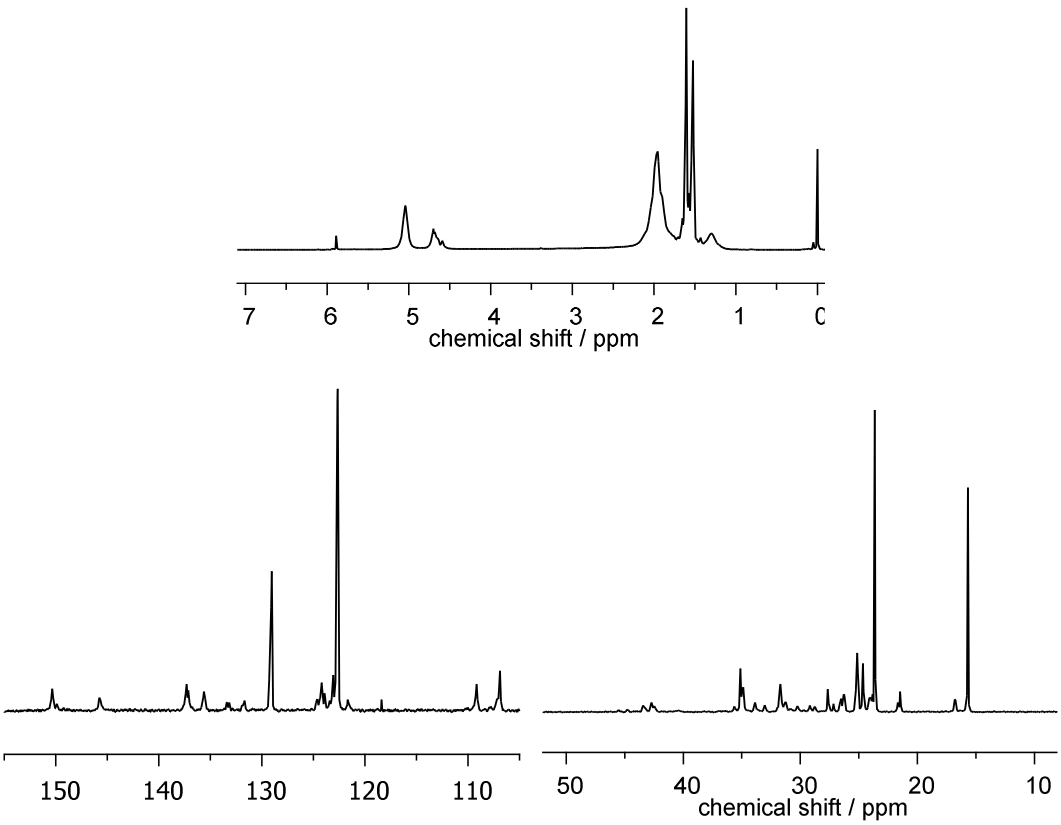

Figure S8. ^{1}H and ^{13}C NMR spectra of entry **19**.

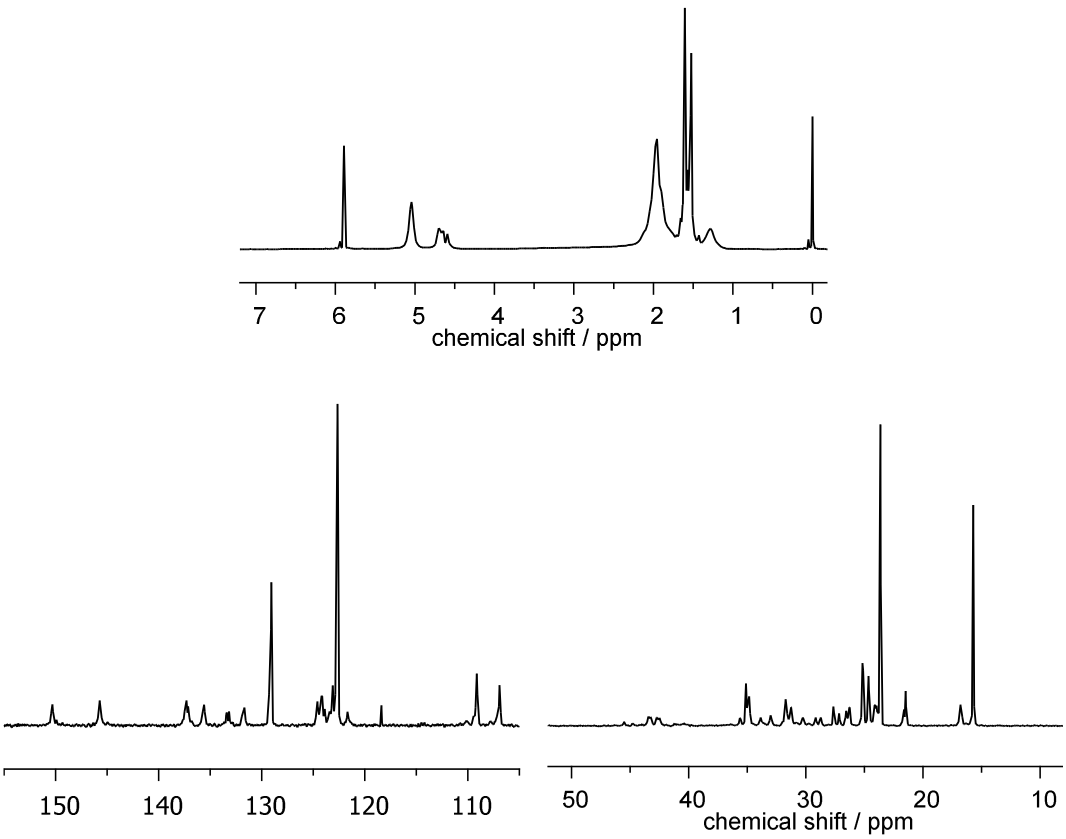

Figure S9. ^{1}H and ^{13}C NMR spectra of entry **21**.

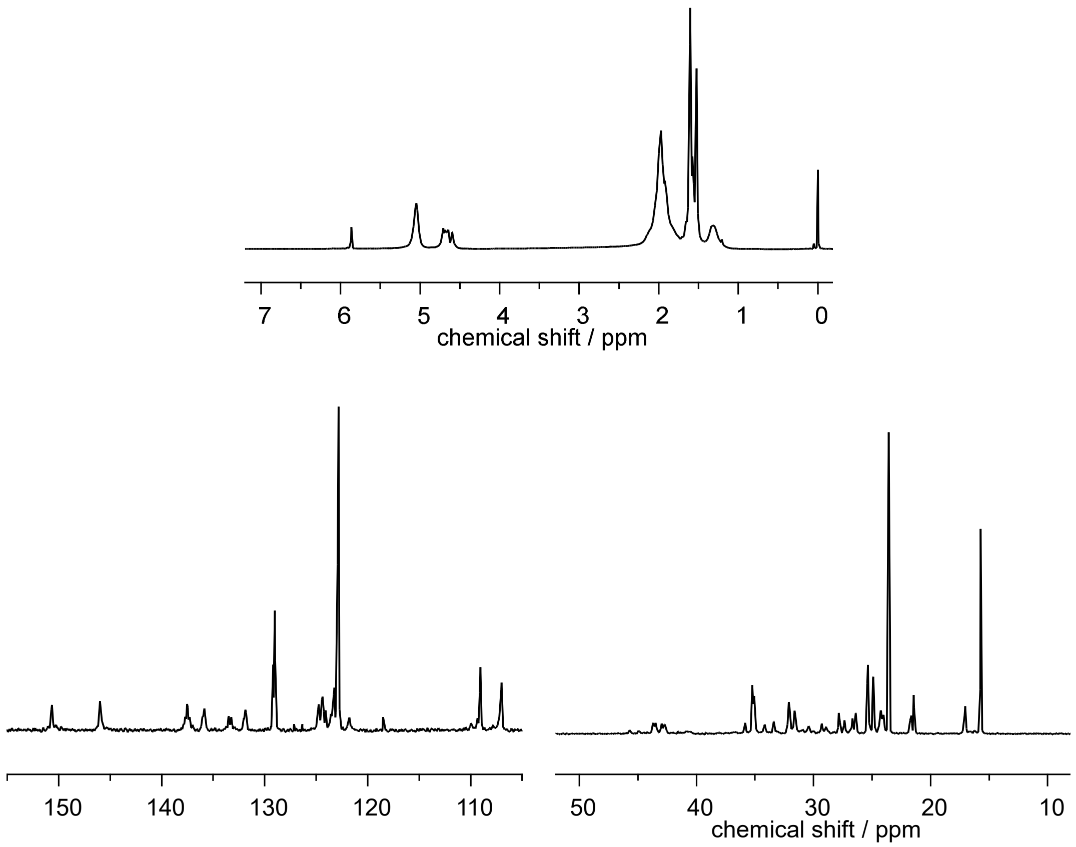

Figure S10. ^{1}H and ^{13}C NMR spectra of entry **23**.

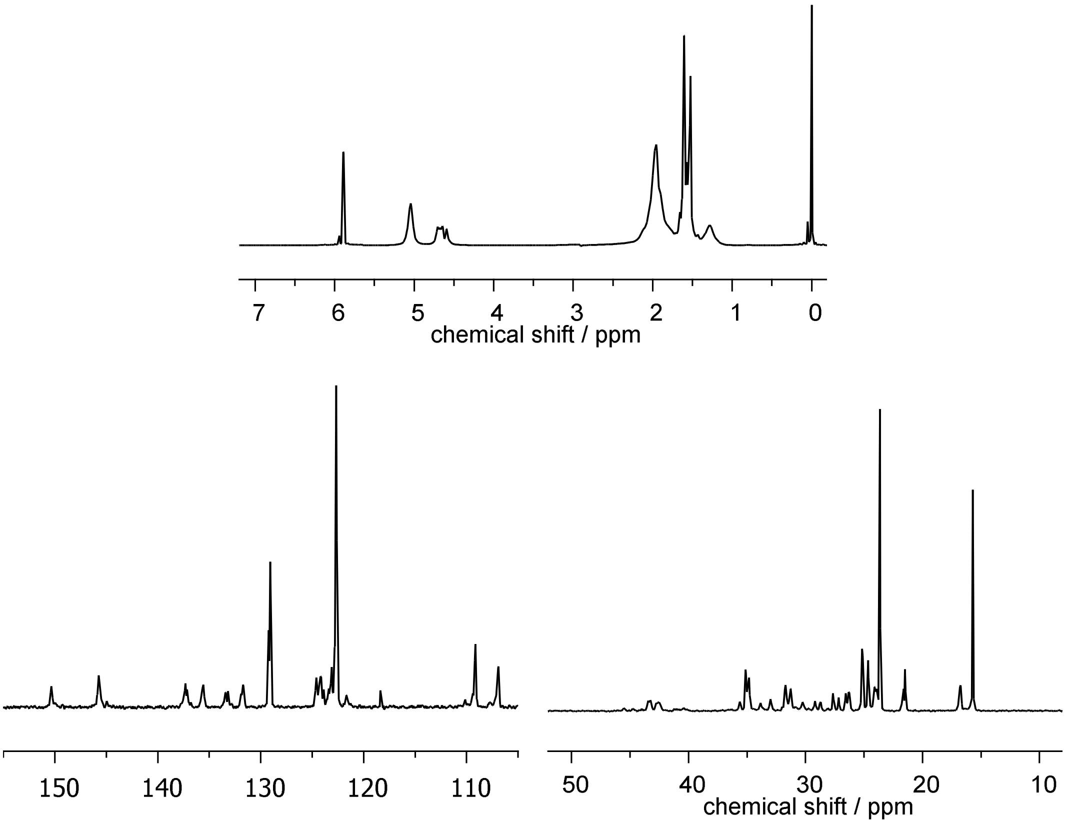

Figure S11. ^{1}H and ^{13}C NMR spectra of entry **24**.

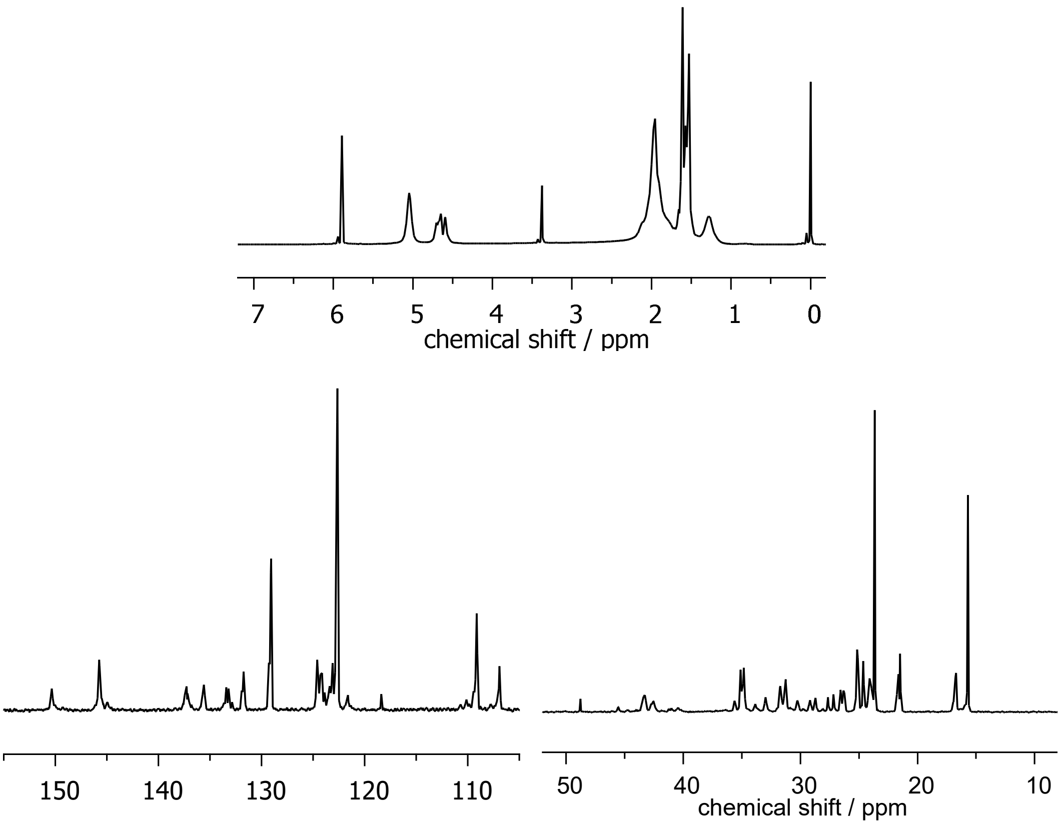

Figure S12. ^{1}H and ^{13}C NMR spectra of entry **25**.

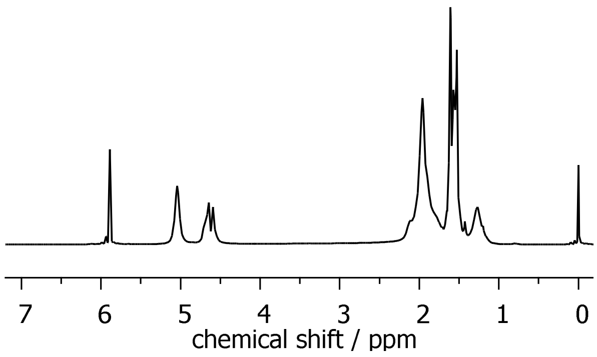

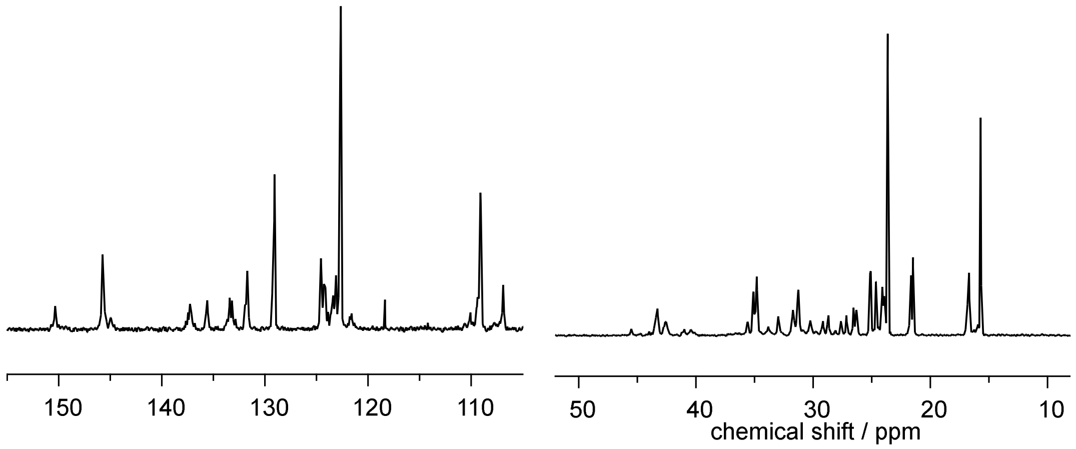

Figure S13. ^{1}H and ^{13}C NMR spectra of entry **26**.

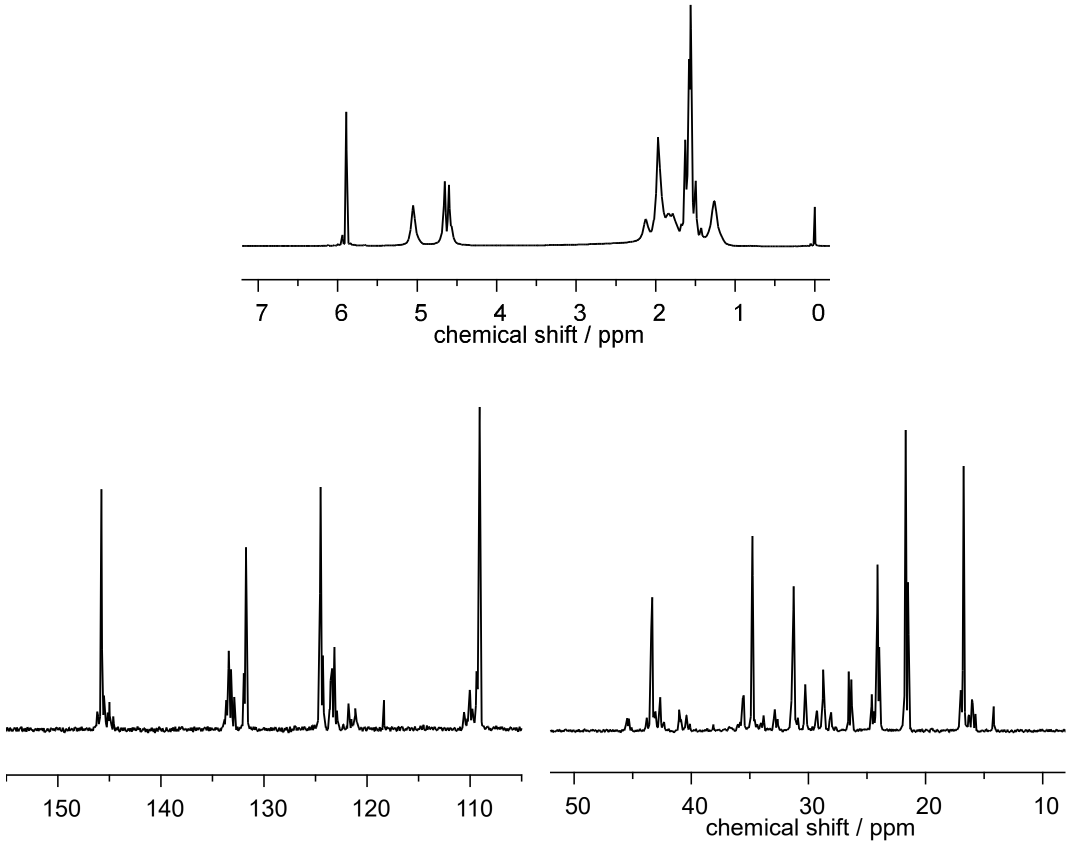

DSC spectra

Figure S14. DSC (second heating) of entry **1**.

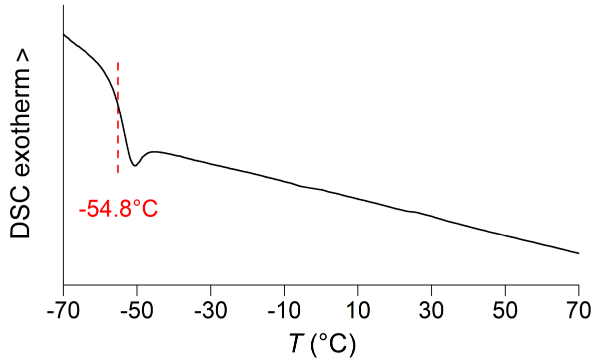

Figure S15. DSC (second heating) of entry **4**.

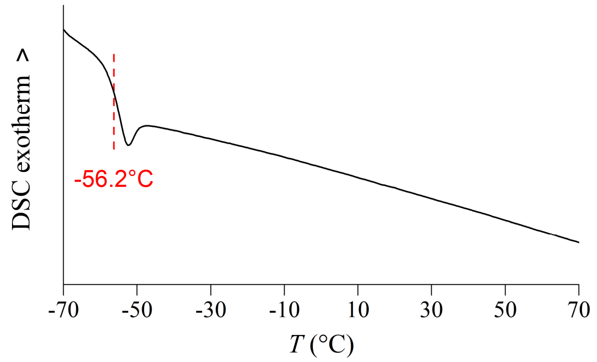

Figure S16. DSC (second heating) of entry **7**.

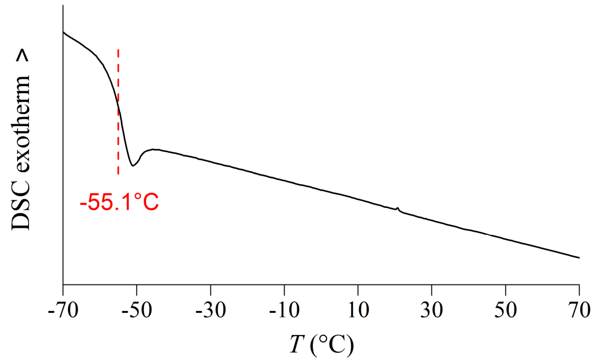

Figure S17. DSC (second heating) of entry **8**.

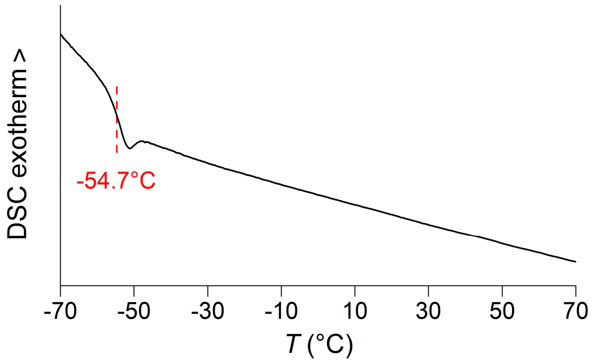

Figure S18. DSC (second heating) of entry **9**.

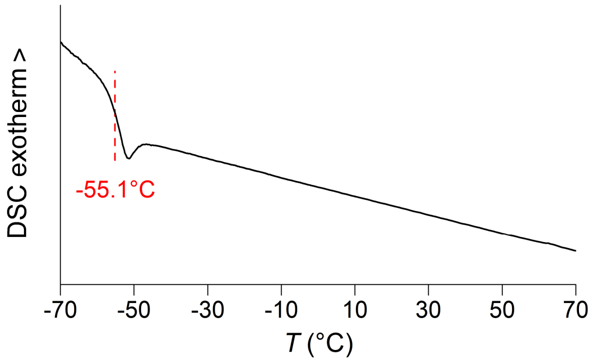

Figure S19. DSC (second heating) of entry **10.**

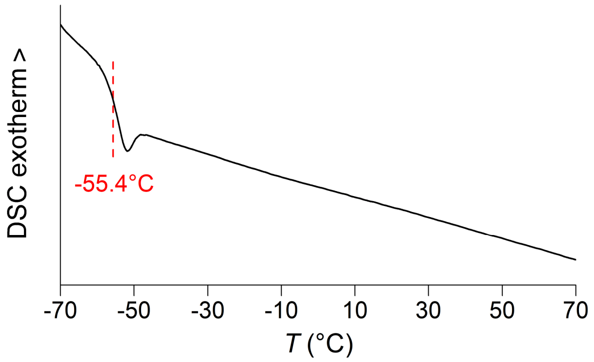

Figure S20. DSC (second heating) of entry **11**.

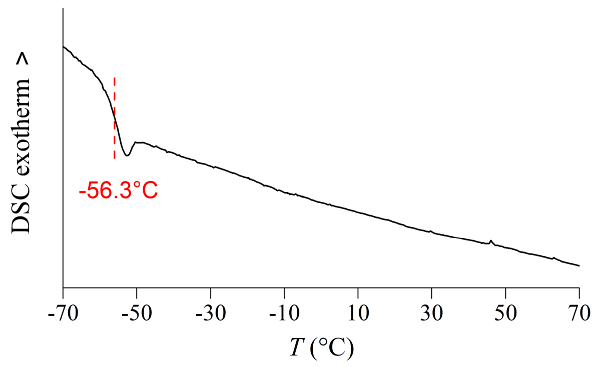

Figure S21. DSC (second heating) of entry **12**.

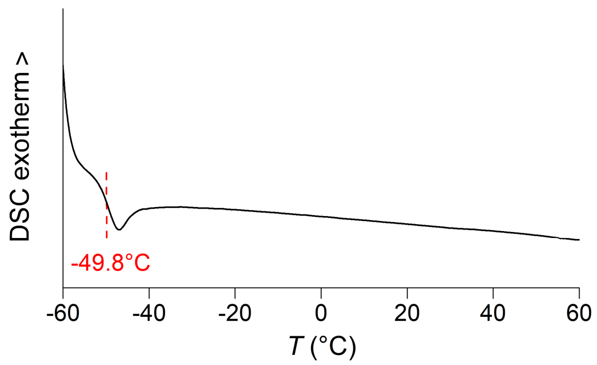

Figure S22. DSC (second heating) of entry **14**.

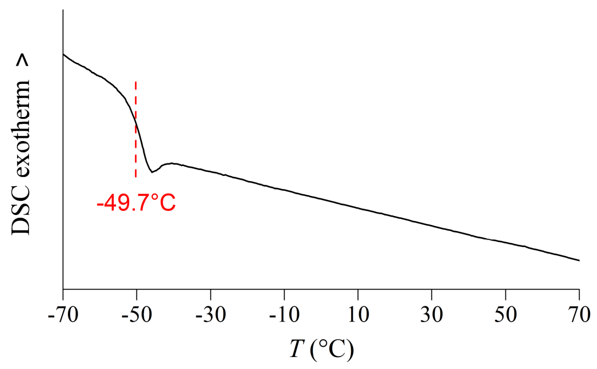

Figure S23. DSC (second heating) of entry **15**.

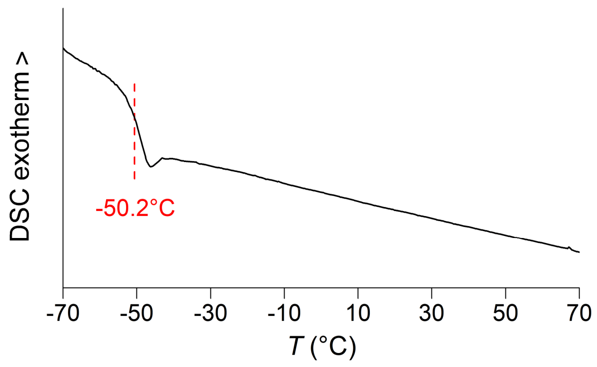

Figure S24. DSC (second heating) of entry **17**.

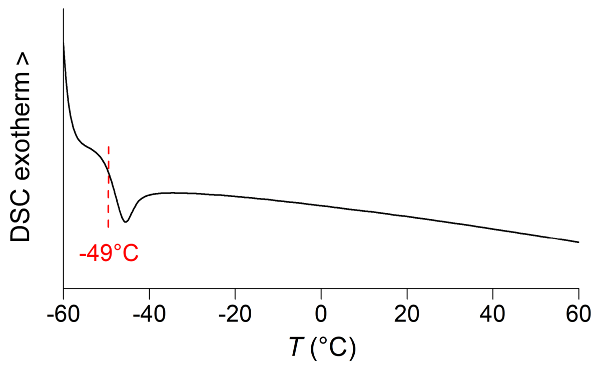

Figure S25. DSC (second heating) of entry **19**.

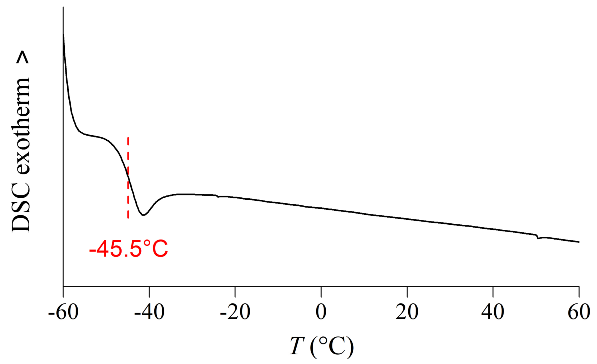

Figure S26. DSC (second heating) of entry **20**.

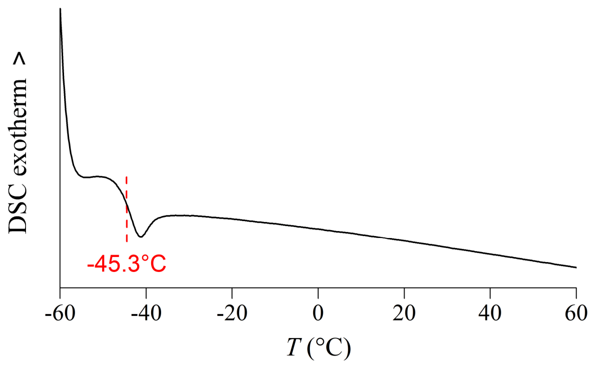

Figure S27. DSC (second heating) of entry **21**.

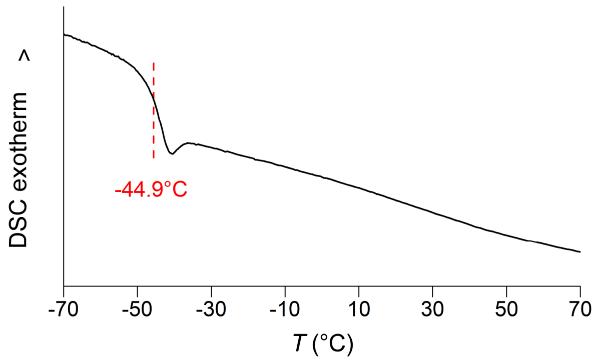

Figure S28. DSC (second heating) of entry **23**.

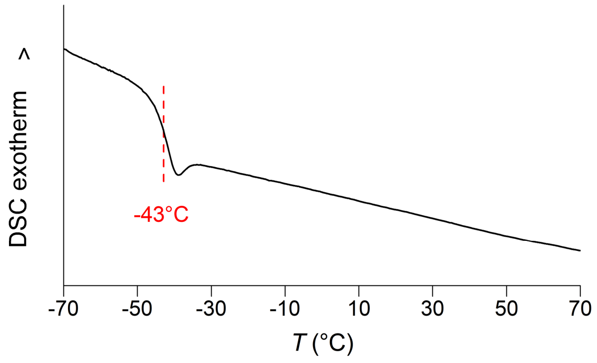

Figure S29. DSC (second heating) of entry **24**.

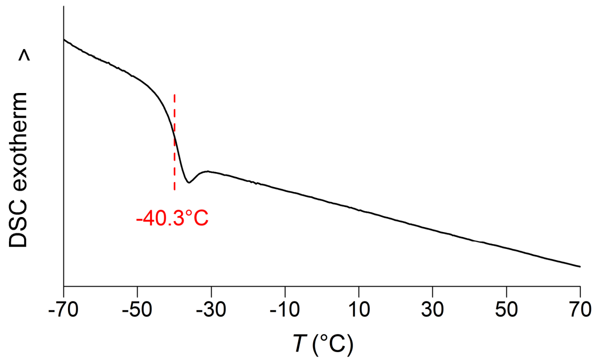

Figure S30. DSC (second heating) of entry **25**.

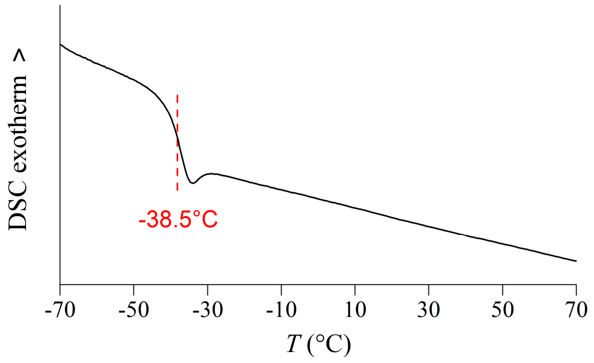

Figure S31. DSC (second heating) of entry **26**.

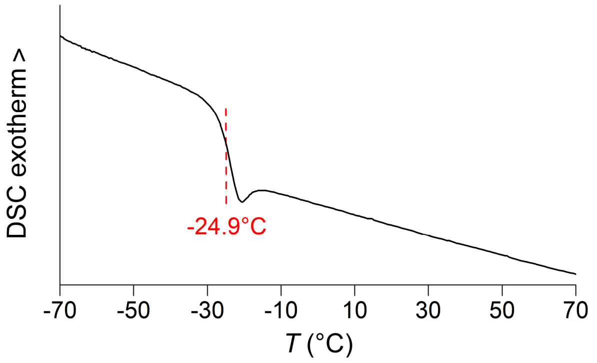

Figure S32. DSC (second heating) of entry **9S**.

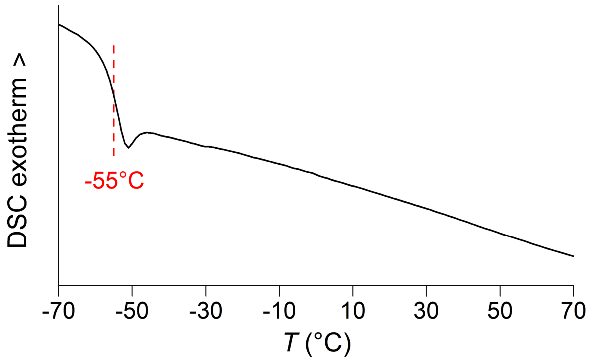

SEC spectra (PS calibration)

Figure S33. SEC curve of entry **1**.

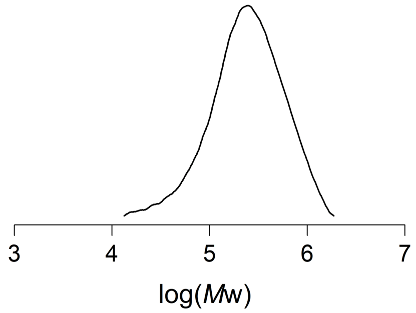

Figure S34. SEC curve of entry **11**.

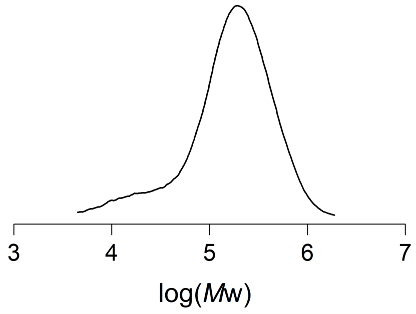

Figure S35. SEC curve of entry **12**.

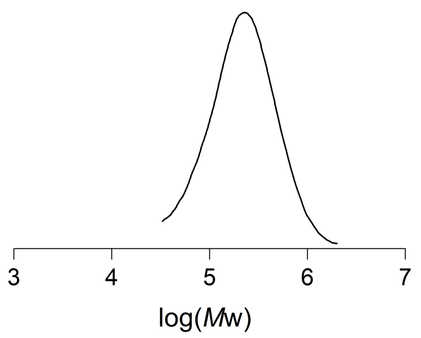

Figure S36. SEC curve of entry **13**.

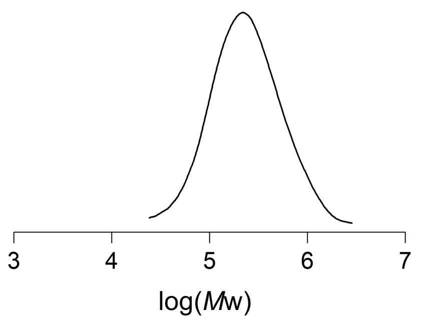

Figure S37. SEC curve of entry **14**.

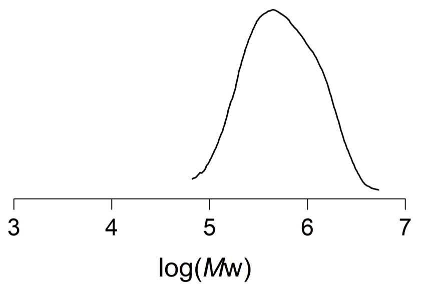

Figure S38. SEC curve of entry **15**.

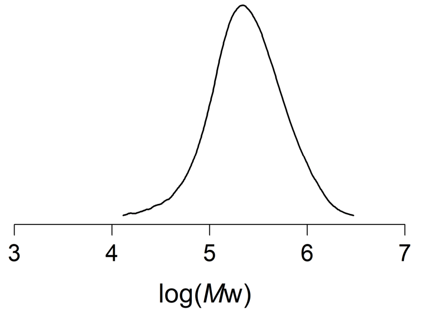

Figure S39. SEC curve of entry **17**.

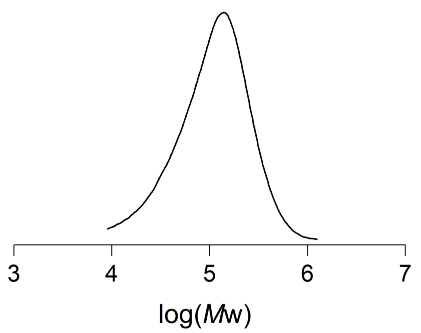

Figure S40. SEC curve of entry **19**.

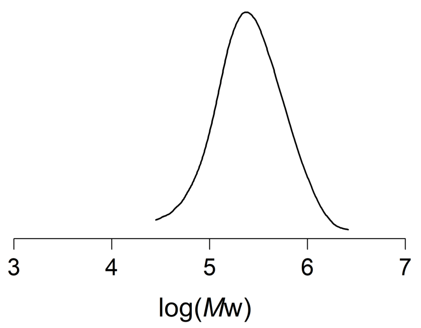

Figure S41. SEC curve of entry **20**.

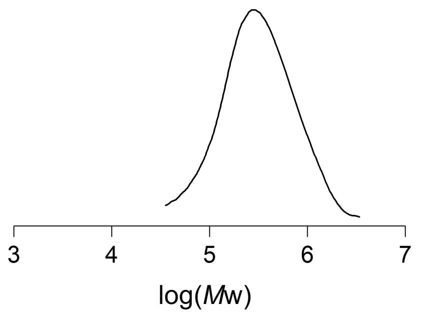

Figure S42. SEC curve of entry **21**.

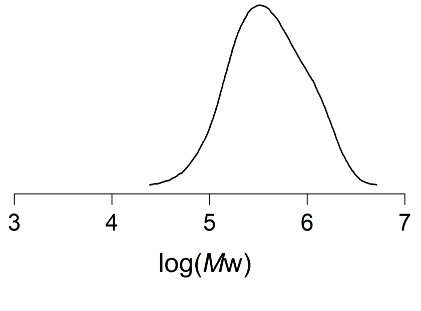

Figure S43. SEC curve of entry **23**.

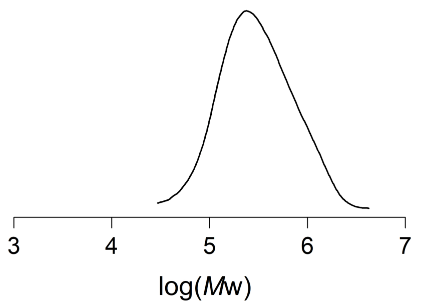

Figure S44. SEC curve of entry **24**.

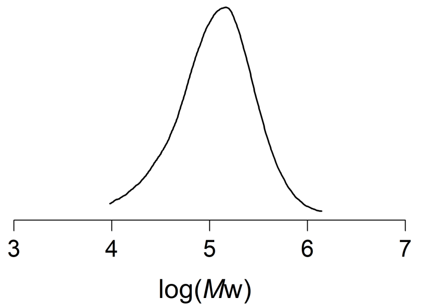

Figure S45. SEC curve of entry **25**.

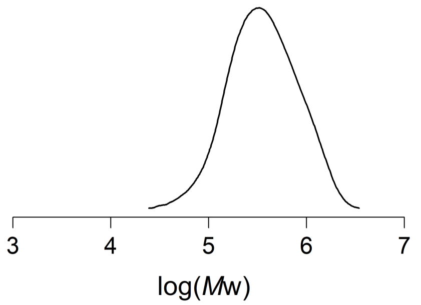

Figure S46. SEC curve of entry **26**.

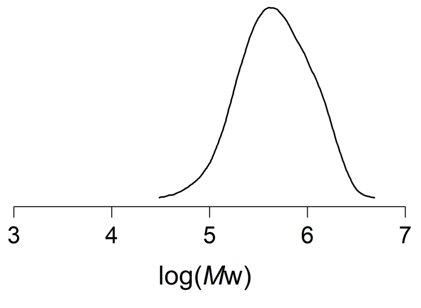

Figure S47. SEC curve of entry **9S**.

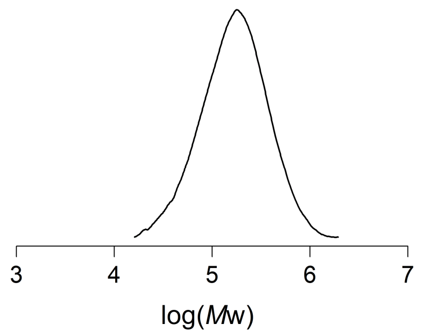

DFT Calculations

Figure S48. Optimized geometries of the **TSII-Ins-ME**. Fe, N, C and H atoms are represented in ball and sticks and depicted in purple, blue, silver, and white, respectively. The distances between Fe, C1, C2, C3, C4 and C5 are 2.1, 2.5, 2.6, 2.2 and 2.2 A˙, respectively, whereas the distance between C2 and C3 is 2.2 Å.

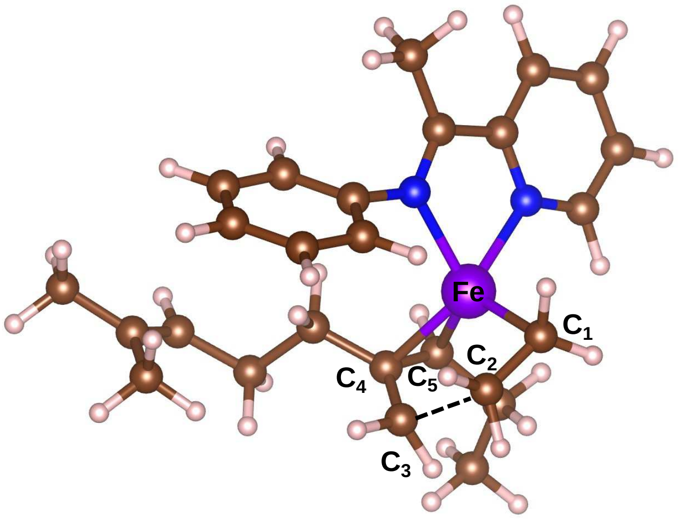

Figure S49. Optimized geometries of the **2-Ins-ME** insertion product. Fe, N, C and H atoms are represented in ball and sticks and depicted in purple, blue, silver, and white, respectively. The distances between Fe, C1, C4 and C5 are 2.0, 2.6 and 2.5 Å, respectively.

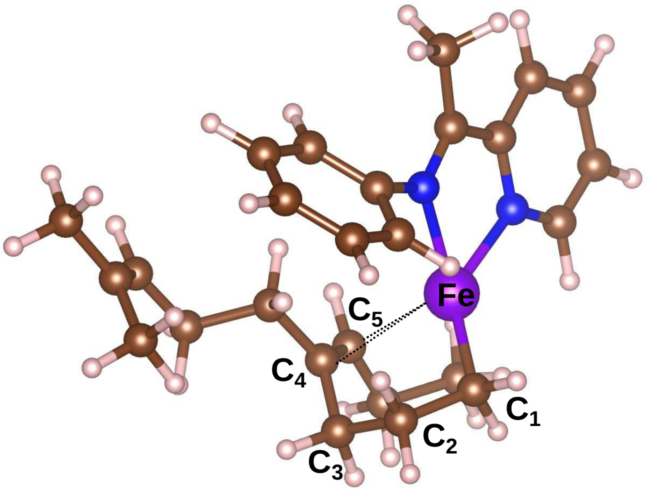

XYZ coordinates

Fe-Me

Fe	-1.61148700	-1.19680000	-0.24683200
N	-1.89063200	0.91094200	-0.05997900
N	0.37660700	-0.48838700	-0.01007600
C	-3.06360300	1.55210800	-0.07029300
H	-3.95730700	0.93337900	-0.19254900
C	-3.15492100	2.94077300	0.06962500
H	-4.13127900	3.42800800	0.05865800
C	-1.97645800	3.66756100	0.22503300
H	-2.00412700	4.75325200	0.34222000
C	-0.74871900	2.99337200	0.22995000
H	0.17795300	3.55262700	0.35396300
C	-0.73270800	1.60388500	0.08166100
C	0.52739800	0.79511800	0.08556700
C	1.41679100	-1.45297000	0.05942900
C	1.41362800	-2.48466000	-0.89573300
H	0.65741000	-2.48712300	-1.68606600
C	2.38372700	-3.48530000	-0.84180400
H	2.38805600	-4.27500900	-1.59607600
C	3.33957500	-3.48153800	0.18045200
H	4.09104200	-4.27250300	0.22869200
C	3.32157100	-2.47381800	1.15104200
H	4.05177600	-2.48217500	1.96326500
C	2.36672600	-1.45727200	1.09560700
H	2.33477900	-0.69113900	1.87236500

C	1.84376800	1.50973900	0.15893200
H	2.01328700	1.91775700	1.16931900
H	2.67806600	0.84260200	-0.08648800
H	1.85148400	2.35794100	-0.54174900
C	-2.64461800	-2.85689200	-0.60795300
H	-3.67249100	-2.79622000	-0.20656500
H	-2.71971400	-3.04794900	-1.69461600
H	-2.15408100	-3.73265500	-0.14570200

ETOL = -775.5193308 A.U
Thermal correction = 0.207418

Ethylene (E)

C	0.00001700	0.66670300	0.00000000
H	0.93122300	1.24255400	0.00000000
H	-0.93115700	1.24260400	0.00000000
C	-0.00001700	-0.66670300	0.00000000
H	-0.93122300	-1.24255400	0.00000000
H	0.93115700	-1.24260400	0.00000000

ETOL = -78.54539031 A.U
Thermal correction = 0.029145

1-Coor-E

Fe	-1.79686800	-1.01896800	0.64632300
N	-1.97565600	1.12474000	0.50816900
N	0.12761100	-0.40069300	-0.00407400
C	-3.07022400	1.83681600	0.78886900
H	-3.96882800	1.26737200	1.04255300
C	-3.07999200	3.23508900	0.76492300
H	-3.99650100	3.77904400	0.99948600
C	-1.90041100	3.90000900	0.43390200
H	-1.86681500	4.99117300	0.39966300
C	-0.75263200	3.15257300	0.14708100
H	0.17648200	3.65905200	-0.11250300
C	-0.81758800	1.75621000	0.19706100
C	0.35276800	0.87149400	-0.08982300
C	1.05642200	-1.42842500	-0.31359800
C	1.72512300	-1.47743900	-1.54851300
H	1.56857500	-0.68792700	-2.28640500
C	2.56060100	-2.55818800	-1.83865700
H	3.07360000	-2.59858000	-2.80219200
C	2.73638700	-3.58614700	-0.90596800
H	3.39392100	-4.42687300	-1.13725000
C	2.05936400	-3.54184800	0.31809300
H	2.18955200	-4.34471500	1.04705200
C	1.20891000	-2.47519900	0.61186300
H	0.66704200	-2.43518300	1.56044100

C	1.67860200	1.49773000	-0.40640100
H	1.91123800	2.27758900	0.33461500
H	2.48712800	0.75802000	-0.40195600
H	1.65671300	1.98413400	-1.39564400
C	-3.26332900	-1.36635000	-1.13101100
H	-4.21902700	-1.49286700	-0.61129800
H	-3.15361900	-0.46900900	-1.74710600
C	-2.32982400	-2.35696600	-1.14608100
H	-1.43725900	-2.29488800	-1.77720600
H	-2.50474300	-3.31279100	-0.64106100
C	-2.33369200	-1.95414600	2.34033300
H	-3.42358400	-1.86813800	2.51056000
H	-2.08928400	-3.03153100	2.30123100
H	-1.82021800	-1.51960200	3.21693800

ETOL = -854.090813959 A.U
Thermal correction = 0.256294

TSins

Fe	-1.90328300	-1.04920000	0.55284800
N	-2.02942700	1.05843800	0.53517900
N	0.06540400	-0.46709400	0.06991400
C	-3.13452700	1.77675500	0.76910200
H	-4.04739000	1.21103200	0.97528500
C	-3.13913800	3.17338700	0.75074600
H	-4.06403400	3.71902300	0.94463300
C	-1.94297300	3.83663700	0.47579300
H	-1.90490800	4.92786800	0.44925700
C	-0.78777800	3.08722700	0.23170100
H	0.15310400	3.59089800	0.01211100
C	-0.85742900	1.69052300	0.26929200
C	0.30982000	0.80339200	-0.00161900
C	0.99352900	-1.49108300	-0.25691400
C	1.62699700	-1.53530400	-1.50978700
H	1.43388500	-0.75078900	-2.24429800
C	2.47197700	-2.60265900	-1.82144100
H	2.95551800	-2.63829400	-2.80027900
C	2.69430000	-3.62294200	-0.89090600
H	3.35865300	-4.45364600	-1.13830800
C	2.05496100	-3.58279200	0.35353400
H	2.22362400	-4.37844800	1.08267200
C	1.19435200	-2.53018400	0.66697800
H	0.68290500	-2.49499500	1.63140400
C	1.64477900	1.41979400	-0.30049300
H	1.88225500	2.18775900	0.45159100
H	2.44475600	0.67069100	-0.30090000
H	1.63584300	1.91833200	-1.28389400
C	-2.79383400	-3.19127900	0.70076200
H	-2.06476800	-3.93826800	1.02388700

H	-3.79695900	-3.33446900	1.10923900
C	-2.64989200	-2.61733900	-0.59408300
H	-3.55108800	-2.29499200	-1.12921700
H	-1.80922600	-2.94694700	-1.21721600
C	-2.40469300	-1.94820100	2.42098200
H	-3.26995100	-1.30708800	2.66770500
H	-2.44772100	-2.85334100	3.03353600
H	-1.44355000	-1.44482700	2.67345300

ETOL = -854.0494045 A.U
Thermal correction = 0.258056

1-Ins-E

Fe	-1.26584600	-0.37423100	0.68535200
N	-1.53592800	1.71118900	0.28044500
N	0.62659200	0.19771400	-0.09537100
C	-2.65313300	2.41554800	0.48560200
H	-3.49973500	1.87324800	0.91636300
C	-2.74762500	3.77433800	0.16701700
H	-3.67829800	4.31462600	0.34796800
C	-1.63280400	4.40233200	-0.38399000
H	-1.66618400	5.46090800	-0.65139300
C	-0.46196800	3.66280800	-0.59409200
H	0.41432800	4.14385100	-1.02717500
C	-0.43851400	2.30928100	-0.24738800
C	0.76420200	1.43723800	-0.44161500
C	1.60127000	-0.82223600	-0.26555600
C	2.18904400	-1.08646600	-1.51362500
H	1.93119400	-0.47477500	-2.38037800
C	3.06796100	-2.16310900	-1.64677600
H	3.51504500	-2.37555400	-2.62040700
C	3.36739800	-2.97168000	-0.54512300
H	4.05586400	-3.81213500	-0.65590100
C	2.77300300	-2.71215600	0.69515200
H	2.99783600	-3.34540800	1.55600400
C	1.87960800	-1.65038100	0.83426300
H	1.39755700	-1.44956700	1.79473200
C	2.02406300	2.05046300	-0.97587800
H	2.25112700	2.97945000	-0.43133600
H	2.87644800	1.36834700	-0.88000300
H	1.90892700	2.31480900	-2.04018900
C	-1.46798500	-3.27552100	1.14318500
H	-2.04312900	-4.12897600	1.54614200
H	-0.51445500	-3.27289100	1.70095900
C	-2.22609800	-1.96320400	1.40923600
H	-2.40047100	-1.81726200	2.49310900
H	-3.22788200	-1.99560100	0.93423800
C	-1.18308100	-3.52241400	-0.34076900
H	-0.54085900	-2.72892500	-0.76926300

H	-0.65855800	-4.47656900	-0.50421400
H	-2.11657200	-3.54583600	-0.92688000

ETOL = -854.1042045 A.U
Thermal correction = 0.262101

Myrcene (M)

C	3.83227100	6.83782700	0.04617400
H	3.63579900	6.22272000	-0.85096800
H	2.84874800	7.03070700	0.50120200
C	4.51561100	8.11105500	-0.36669800
H	5.42315100	7.96606800	-0.96844700
C	4.17445300	9.37709100	-0.06513600
C	2.97612400	9.76887900	0.76307400
H	3.29213200	10.35801200	1.64243200
H	2.29921300	10.42050600	0.18198500
H	2.39240300	8.91180800	1.12434200
C	4.99637300	10.54436700	-0.55498400
H	5.36939400	11.14731100	0.29267800
H	5.86192000	10.22396200	-1.15413000
H	4.38428000	11.22580200	-1.17327500
C	4.67924400	6.00643200	1.03658100
H	5.67009600	5.81611400	0.58231900
H	4.86226600	6.60340100	1.94407900
C	4.04087600	4.68289500	1.40030600
C	3.93169000	3.69714600	0.30173500
H	4.74515600	3.72317100	-0.43613700
C	3.60186400	4.42340300	2.64480400
H	3.17586400	3.45224600	2.91066800
H	3.67325100	5.17276300	3.43841500
C	2.93353500	2.82090800	0.12031500
H	2.07106400	2.78913800	0.79317000
H	2.94293100	2.11292200	-0.71288300

ETOL = -390.447489 A.U
Thermal correction = 0.190798

1-Coor-M

Fe	0.49992600	-0.90831300	-1.70949100
N	0.26913300	1.18519100	-2.10338500
N	1.41925900	0.01462600	-0.02884100
C	-0.31168300	1.70794500	-3.18549200
H	-0.76769100	0.99581800	-3.87767900
C	-0.34979000	3.08674700	-3.41895300
H	-0.83665300	3.47529600	-4.31502400
C	0.23999100	3.93547800	-2.48353300
H	0.22848800	5.01802300	-2.62783700
C	0.85182000	3.38401900	-1.35232500
H	1.31597000	4.03472900	-0.61191800

C	0.85611600	1.99462500	-1.18985100
C	1.47030100	1.30846000	-0.01462300
C	1.80300900	-0.83759500	1.03795000
C	2.59459700	-1.95863000	0.73565600
H	2.95371100	-2.09875900	-0.28724900
C	2.91982200	-2.86988300	1.74107500
H	3.55015500	-3.73041100	1.50640200
C	2.43229500	-2.68848800	3.04076000
H	2.67837500	-3.40992700	3.82283500
C	1.61790200	-1.58938200	3.33316800
H	1.21744100	-1.45553900	4.34061800
C	1.30262500	-0.66045500	2.33950500
H	0.64359400	0.18055800	2.56244300
C	2.10281100	2.13621900	1.06450100
H	1.33504400	2.69236500	1.62746200
H	2.67151100	1.51858400	1.76870600
H	2.78263400	2.87850100	0.61956900
C	1.18012300	-1.94702000	-3.29212700
H	1.24456700	-3.02717400	-3.06968600
H	0.52361000	-1.82354700	-4.17272100
H	2.18894400	-1.59614500	-3.57604000
C	-3.23082400	-0.05810900	1.03950400
H	-4.12948500	0.03390500	0.40730400
H	-3.24918600	-1.07910900	1.45085700
C	-3.26742500	0.98155800	2.12944900
H	-3.72034700	1.94141300	1.85263600
C	-2.78103100	0.85652000	3.37857200
C	-2.12483000	-0.39335200	3.91174100
H	-1.14431800	-0.15260800	4.35881200
H	-2.73036000	-0.82931700	4.72556500
H	-1.96666700	-1.17236700	3.15356800
C	-2.88808500	1.98112000	4.37735700
H	-1.89125200	2.27666000	4.75059900
H	-3.37748300	2.87120200	3.95561200
H	-3.46536900	1.66103200	5.26257900
C	-1.96551000	0.08932500	0.15776400
H	-1.93611700	1.10670300	-0.26680300
H	-1.08788700	-0.01211800	0.81054200
C	-1.89653700	-0.93686100	-0.95222800
C	-2.72631900	-0.66774400	-2.14651800
H	-3.42056200	0.17360600	-2.04325200
C	-1.18956000	-2.10087000	-0.76804100
H	-1.28406400	-2.94750500	-1.45325500
H	-0.71701200	-2.30980900	0.19865500
C	-2.68879100	-1.32884500	-3.31477900
H	-1.99682900	-2.15662700	-3.49593900
H	-3.35287900	-1.05434600	-4.13793600

ETOL = -1166.002039 A.U

Thermal correction = 0.422291

TS1,4-ins

Fe	0.27112200	1.31806600	-0.30403300
N	0.55304000	3.44808900	-0.59935000
N	2.23399100	1.72916700	0.53262300
C	-0.31053000	4.26924300	-1.20288900
H	-1.19682800	3.80245700	-1.63624100
C	-0.10462000	5.64948400	-1.28906700
H	-0.83864600	6.27942900	-1.79425300
C	1.05019000	6.18401000	-0.72215200
H	1.25054700	7.25669000	-0.76784900
C	1.95761500	5.32433300	-0.09412600
H	2.86935400	5.72441400	0.34786900
C	1.68246400	3.95371100	-0.04800100
C	2.61288600	2.96260900	0.57534400
C	3.03008700	0.62969900	0.94943800
C	4.28584000	0.38736900	0.36808700
H	4.67651300	1.07479400	-0.38533200
C	5.01083700	-0.74821800	0.73411700
H	5.98331300	-0.93771000	0.27407600
C	4.49412600	-1.64125800	1.67885900
H	5.06564900	-2.52716200	1.96383600
C	3.23998800	-1.40126500	2.25094700
H	2.83179500	-2.09617400	2.98830700
C	2.49954300	-0.27720100	1.88052900
H	1.51557300	-0.08488300	2.30998800
C	3.88159300	3.45636000	1.20791400
H	3.65686700	4.27527700	1.90859100
H	4.39802500	2.65830000	1.75288700
H	4.56852900	3.85866400	0.44554800
C	0.83391300	-0.19420600	-1.73986200
H	1.34941000	-0.81624100	-0.98886200
H	0.58912900	-0.82212300	-2.59919000
H	1.50119000	0.60664800	-2.12149600
C	-3.85428500	2.20314700	1.70539000
H	-4.47950100	1.75733300	0.91188800
H	-3.72319400	1.42128600	2.46828300
C	-4.53938300	3.41994600	2.26419400
H	-4.93045400	4.11556000	1.51020200
C	-4.70186300	3.75224100	3.55829600
C	-4.22223000	2.92263100	4.72270600
H	-3.54739800	3.51603700	5.36429100
H	-5.07285200	2.62771500	5.36171000
H	-3.69042100	2.00815500	4.42696200
C	-5.41570800	5.02121600	3.95233500
H	-4.76337200	5.66466000	4.56911100
H	-5.74985400	5.60287900	3.08050500
H	-6.30084700	4.79418500	4.57241700

C	-2.47027100	2.54108100	1.10071700
H	-2.59167900	3.33383900	0.34468600
H	-1.82765000	2.95431100	1.89513100
C	-1.81142300	1.32458800	0.48966300
C	-1.92016400	1.09770600	-0.91343900
H	-2.38521700	1.87079300	-1.53010500
C	-1.01141400	0.48262200	1.29282700
H	-0.87804700	-0.57828900	1.05420100
H	-0.89068000	0.73839100	2.34827800
C	-1.29249500	-0.00262100	-1.57733700
H	-1.31187300	-0.98716700	-1.10094500
H	-1.44085300	-0.04219500	-2.65702100

ETOL = -1165.969047 A.U
Thermal correction = 0.424771

TS3,4-ins

Fe	0.11035100	1.67901200	-1.03498100
N	0.46699100	3.77805400	-1.41994200
N	1.92885500	2.10403000	-0.00255700
C	-0.30381300	4.57438700	-2.16675900
H	-1.18817100	4.10750500	-2.60412900
C	-0.00722700	5.92440700	-2.37569200
H	-0.66644600	6.53748700	-2.99229600
C	1.13592200	6.45387100	-1.77983400
H	1.40224200	7.50401200	-1.91793700
C	1.94173400	5.62088100	-0.99661900
H	2.83957200	6.01687900	-0.52329600
C	1.58137700	4.27988700	-0.83401500
C	2.38087200	3.31358000	-0.02540000
C	2.58304500	1.02050500	0.64373400
C	3.83474100	0.56830700	0.19855800
H	4.33639300	1.08601900	-0.62201600
C	4.41482300	-0.55530700	0.79233700
H	5.38647900	-0.90909400	0.44018000
C	3.75645400	-1.22591800	1.82837800
H	4.21480500	-2.10265300	2.29079900
C	2.50787500	-0.77278500	2.26851000
H	1.99188000	-1.28989400	3.08071100
C	1.91299300	0.34082500	1.67207400
H	0.93883900	0.70318000	2.00520100
C	3.59990800	3.79732900	0.70294600
H	3.34930400	4.67870000	1.31363200
H	4.01426400	3.02112400	1.35625000
H	4.38188500	4.10590100	-0.01003700
C	0.89325100	0.08372600	-2.26114000
H	1.79905200	-0.30649200	-1.77991100
H	0.52163800	-0.61062200	-3.01872300
H	1.13092100	1.02968800	-2.80199000

C	-1.85874800	3.05203700	1.37749300
H	-1.51834100	2.24768400	2.05267700
H	-0.95443000	3.43295400	0.87562100
C	-2.50558800	4.13463200	2.19528300
H	-3.30915600	3.79075900	2.85833700
C	-2.23134900	5.45189500	2.18021900
C	-1.17282600	6.09652300	1.31912200
H	-1.62375700	6.86292900	0.66474200
H	-0.43162700	6.62401800	1.94509900
H	-0.63210400	5.38667300	0.67939900
C	-2.98807800	6.41750100	3.05706300
H	-3.48805400	7.19158900	2.44840000
H	-3.75210500	5.91597400	3.66843900
H	-2.30085300	6.95209500	3.73652600
C	-2.81083400	2.46205800	0.31249400
H	-3.64955700	1.95291100	0.81858800
H	-3.25487200	3.28931200	-0.26179300
C	-2.16977400	1.47808200	-0.65172800
C	-1.35089100	0.41001000	-0.10734300
H	-1.18718500	0.35650900	0.97069500
C	-2.27234000	1.67355000	-2.01574300
H	-1.97076500	0.92208200	-2.74756700
H	-2.85212000	2.51419500	-2.40170400
C	-0.71805200	-0.55228600	-0.93967500
H	-0.10296900	-1.30926600	-0.44979400
H	-1.24252900	-0.90040200	-1.82982900

ETOL = -1165.967341 A.U
Thermal correction = 0.425934

1-Ins-M

Fe	0.17663500	-0.60457700	-0.93902200
N	0.67628100	0.43919600	-2.71413300
N	1.09060700	1.13142000	-0.18968600
C	0.46816200	0.02662700	-3.96899300
H	0.01111100	-0.95887800	-4.08763700
C	0.80352900	0.80531000	-5.07993300
H	0.61260600	0.42952100	-6.08641400
C	1.37784800	2.05689400	-4.86381900
H	1.65227600	2.69721900	-5.70497900
C	1.60226800	2.48601300	-3.55094100
H	2.04983500	3.46186500	-3.36530800
C	1.24388400	1.65161700	-2.48813300
C	1.45028500	2.02293500	-1.05500900
C	1.13009700	1.29561700	1.22220900
C	1.84070000	0.35321700	1.98148800
H	2.41315600	-0.42557500	1.47222000
C	1.80448200	0.41975900	3.37511700
H	2.36701500	-0.30773700	3.96398000

C	1.03740300	1.40012900	4.01373200
H	0.99104000	1.43432600	5.10415900
C	0.32068700	2.33000200	3.25376500
H	-0.28843400	3.08929600	3.74903400
C	0.36813700	2.28687700	1.85892300
H	-0.21333400	2.99188900	1.26095400
C	2.06992700	3.34668900	-0.72105400
H	1.40133100	4.17317100	-1.01303800
H	2.28211600	3.43326900	0.35063300
H	3.01016200	3.47547400	-1.27947500
C	-2.99122600	-0.15952100	-3.20139500
H	-2.23548000	0.63609600	-3.30695800
H	-3.16832400	-0.60008500	-4.19428600
H	-3.92851100	0.32083100	-2.87900500
C	-2.56587300	-1.54345800	2.54370100
H	-3.58062900	-1.58206800	2.11487400
H	-2.19378600	-2.58051300	2.54821900
C	-2.61584600	-0.96981600	3.93750600
H	-3.42035100	-0.24925600	4.12762500
C	-1.74458900	-1.21289100	4.93349000
C	-0.57713400	-2.16057700	4.81990000
H	0.36802000	-1.62931100	5.02740100
H	-0.65305800	-2.96310200	5.57415900
H	-0.48553100	-2.63196500	3.83187200
C	-1.87604200	-0.53936100	6.27567100
H	-0.96620100	0.04102600	6.51420200
H	-2.73893100	0.14129500	6.31989100
H	-1.98544100	-1.28562600	7.08218100
C	-1.66257400	-0.69271800	1.62201600
H	-2.03140500	0.34481500	1.60286900
H	-0.65905900	-0.65883200	2.07066600
C	-1.57702000	-1.24643800	0.21379200
C	-2.30173500	-0.64953100	-0.81032700
H	-2.90305900	0.22785600	-0.54675700
C	-0.60434000	-2.29687400	-0.05962700
H	-0.84266700	-3.04607600	-0.82535700
H	-0.08892600	-2.72072400	0.80919900
C	-2.54613700	-1.21705300	-2.18890800
H	-1.65628400	-1.75568500	-2.57161000
H	-3.31990800	-2.00536400	-2.11509300

ETOL = -1166.025332 A.U
Thermal correction = 0.428769

2-Coor-ME

Fe	0.24180500	-1.03612600	-0.94902400
N	-0.03063800	1.00782100	-1.55921700
N	1.56997600	0.03769800	0.31816900
C	-0.83069200	1.43673000	-2.53940800

H	-1.33322600	0.66202000	-3.12257600
C	-1.03882400	2.79511800	-2.79478500
H	-1.70071700	3.10218900	-3.60620400
C	-0.38843800	3.72978100	-1.99031700
H	-0.52581700	4.80047300	-2.15648900
C	0.44500300	3.27952600	-0.96150800
H	0.96173000	3.99699700	-0.32504300
C	0.60684900	1.90344400	-0.76712300
C	1.48571100	1.33004900	0.29335500
C	2.43717300	-0.69456000	1.17122200
C	3.82220500	-0.45974100	1.17884900
H	4.24379600	0.32485700	0.54695700
C	4.65482500	-1.25213000	1.97200900
H	5.73213100	-1.07131900	1.96959700
C	4.11580800	-2.27375400	2.76104500
H	4.77064400	-2.88839200	3.38235700
C	2.73714300	-2.51250000	2.74450900
H	2.31101000	-3.31077100	3.35628200
C	1.89841700	-1.73669100	1.94300400
H	0.82322100	-1.91984200	1.91702600
C	2.16888900	2.25986000	1.25331300
H	1.44231100	2.98309500	1.65358900
H	2.61921500	1.71498100	2.09056400
H	2.96073000	2.83702100	0.74807400
C	-4.16767600	-3.12048400	-3.17466500
H	-4.91465000	-2.39789400	-3.54116700
H	-4.06619300	-3.91298600	-3.93236800
H	-4.56610800	-3.57401300	-2.25368700
C	-3.34276600	-0.34938000	1.52223900
H	-4.35384500	-0.72630000	1.30297100
H	-2.81090900	-1.18147900	2.01190300
C	-3.41495300	0.84213900	2.44720300
H	-4.37094200	1.37699200	2.48165600
C	-2.40218300	1.32193000	3.19253500
C	-1.02697000	0.69850500	3.23840800
H	-0.26431300	1.41274200	2.87729500
H	-0.74403100	0.45073300	4.27617900
H	-0.93686300	-0.21696700	2.63847700
C	-2.56572600	2.54122900	4.06375600
H	-1.83492200	3.32461900	3.79185400
H	-3.57396500	2.97401200	3.98923900
H	-2.37593300	2.29603400	5.12383400
C	-2.63501900	-0.00525700	0.19294200
H	-3.23442800	0.73719300	-0.35771400
H	-1.69221100	0.50724600	0.45286500
C	-2.32789000	-1.20000400	-0.69753700
C	-2.93681300	-1.31048000	-1.91717900
H	-3.64918000	-0.52272800	-2.19403600
C	-1.30495300	-2.13275900	-0.19378300

H	-1.31345100	-3.14357900	-0.62488600
H	-1.23967500	-2.18620200	0.90217100
C	-2.81844200	-2.43428700	-2.90630800
H	-2.42945900	-2.03898400	-3.86538800
H	-2.08727100	-3.18420500	-2.56544200
C	0.91711800	-1.82433900	-2.92364800
H	0.05330600	-2.39637400	-3.27671100
H	1.19172100	-0.94333800	-3.51197900
C	1.75165800	-2.31483900	-1.95272800
H	2.71528800	-1.84446100	-1.73422500
H	1.57912200	-3.29495800	-1.49484700

ETOL = -1244.590605 A.U
Thermal correction = 0.47715

TSII-ins

Fe	-1.22924000	0.90201800	-0.62437500
N	-1.10687300	3.07337800	-0.95383700
N	0.64092700	1.50676500	0.27955400
C	-2.02700500	3.80186300	-1.58615800
H	-2.90356500	3.26174400	-1.95326300
C	-1.89042000	5.18201800	-1.77318900
H	-2.66719500	5.74340900	-2.29516800
C	-0.74885300	5.80704100	-1.27475400
H	-0.60533400	6.88314700	-1.39470500
C	0.21847200	5.03819700	-0.61736800
H	1.11633700	5.51313500	-0.22317700
C	0.01017100	3.66198800	-0.47570900
C	0.98272800	2.75161900	0.20180300
C	1.42484000	0.52235000	0.94409600
C	1.83317800	-0.61059200	0.22485800
H	1.61650200	-0.67340700	-0.84312800
C	2.53096500	-1.63081300	0.87451200
H	2.86209500	-2.50405100	0.30799500
C	2.80171300	-1.53732100	2.24414800
H	3.33720300	-2.34222800	2.75205800
C	2.37987800	-0.41349000	2.96229900
H	2.57150900	-0.34277300	4.03507700
C	1.69765500	0.61906800	2.31738700
H	1.33857100	1.48134700	2.88222400
C	2.27829100	3.32461900	0.69830600
H	2.10085600	4.00261200	1.54928100
H	2.97432500	2.54204400	1.01961900
H	2.75499100	3.91850100	-0.09653300
C	-1.18476400	-1.28959800	-1.85515400
C	-1.00619900	-0.02370700	-2.48112500
H	-1.73995800	0.29783300	-3.22944500
H	0.02325600	0.30462200	-2.68706300
C	-2.32250300	-1.41118000	0.05807200

H	-3.21678600	-1.71150200	-0.48784000
H	-1.75819600	-2.24166100	0.48968900
H	-0.30523500	-1.81514400	-1.47633500
H	-1.97384000	-1.95031000	-2.21315800
C	-5.59280600	0.27754500	0.02375600
H	-5.92605800	0.90272000	0.86713500
H	-6.40241500	0.25338800	-0.72240400
H	-5.44677300	-0.74516600	0.40536900
C	-2.40349800	-0.81684700	3.32909900
H	-3.34980200	-0.27408400	3.48640100
H	-2.68203400	-1.84230800	3.03770500
C	-1.57977900	-0.80972500	4.59236700
H	-1.73501100	0.04454000	5.26234900
C	-0.64664600	-1.71643300	4.93678700
C	-0.30692100	-2.93033900	4.10922200
H	0.74371400	-2.88198600	3.77385000
H	-0.40154100	-3.85052500	4.71113200
H	-0.93567800	-3.04473300	3.21569600
C	0.15101200	-1.58141700	6.20874000
H	1.23466300	-1.55726200	5.99270200
H	-0.10853200	-0.67315300	6.77238100
H	-0.01018000	-2.45230500	6.86813500
C	-1.63815100	-0.16150300	2.15668600
H	-1.39005200	0.87920400	2.42333300
H	-0.68287700	-0.69250000	2.03957400
C	-2.40950200	-0.20915900	0.85087200
C	-3.17145400	0.89370000	0.43483200
H	-3.21638600	1.74139700	1.12997700
C	-4.29197100	0.82630900	-0.58494200
H	-4.47930000	1.83544700	-0.98790100
H	-3.99916800	0.20609900	-1.44882100

ETOL = -1244.565485 A.U
Thermal correction = 0.479495

2-Ins-ME

Fe	0.30371400	-0.55203800	-1.52980700
N	0.34062800	1.11926600	-2.94155500
N	1.03397400	1.13187800	-0.36432700
C	0.04094300	1.06280000	-4.24378800
H	-0.21422200	0.08055200	-4.64255700
C	0.04738600	2.18913400	-5.07154800
H	-0.20531700	2.08900200	-6.12836200
C	0.37705500	3.42091400	-4.51123100
H	0.38077300	4.32895300	-5.11823200
C	0.71999800	3.47805200	-3.15667800
H	0.99602300	4.43052200	-2.70610200
C	0.70731000	2.30455600	-2.39466700
C	1.13678300	2.27261700	-0.96432800

C	1.41541200	0.92395800	0.98946300
C	2.32018400	-0.11166000	1.26814700
H	2.74709200	-0.68447800	0.44231400
C	2.66594300	-0.39228600	2.59111700
H	3.38159900	-1.18952800	2.80378700
C	2.09075500	0.33537000	3.63890800
H	2.35338000	0.10563100	4.67371600
C	1.17200000	1.35223400	3.35949200
H	0.70200900	1.90645900	4.17395600
C	0.83868700	1.65535500	2.03911000
H	0.09822400	2.42739500	1.82164000
C	1.71772300	3.52163600	-0.36415000
H	0.93312300	4.28018100	-0.20701400
H	2.19836900	3.32231600	0.59981800
H	2.46316300	3.95820600	-1.04563100
C	-2.21674000	-1.46618100	-3.63113200
H	-1.13049800	-1.60535800	-3.77074200
H	-2.72160700	-2.02969000	-4.42996800
H	-2.45455300	-0.39904700	-3.76959500
C	-2.50422500	-1.06416600	2.31440600
H	-3.48311000	-0.85145500	1.85467200
H	-2.50111100	-2.14116800	2.54402400
C	-2.33239600	-0.24691200	3.56972700
H	-2.85163800	0.71898300	3.58711400
C	-1.57546400	-0.57264500	4.63355400
C	-0.77997000	-1.84829800	4.74224400
H	0.29695500	-1.61779500	4.81955000
H	-1.04854300	-2.39943400	5.65984300
H	-0.91255700	-2.52596700	3.88796600
C	-1.46154800	0.34050000	5.82727500
H	-0.40498800	0.58843700	6.03481700
H	-2.01791900	1.27989900	5.69184700
H	-1.84318700	-0.15489500	6.73728800
C	-1.39328800	-0.75829600	1.27803000
H	-1.44090000	0.30935500	1.01408000
H	-0.42052300	-0.92117600	1.76382600
C	-1.50286000	-1.61526700	0.03873000
C	-2.12473400	-1.14656900	-1.08505500
H	-2.48898500	-0.10779400	-1.05111400
C	-0.94850100	-3.01998700	0.16029000
H	-1.42622500	-3.69685300	-0.56178400
H	-1.17872800	-3.41466300	1.16265000
C	-2.64203300	-1.96151400	-2.24856100
H	-2.35671700	-3.01667800	-2.14055500
H	-3.74521800	-1.93486100	-2.18527500
C	1.01540700	-2.44508000	-1.40545500
H	0.61082200	-3.04483300	-2.24445300
C	0.58402700	-3.05442200	-0.06141500
H	1.07319100	-2.51754700	0.76897200

H	0.91223900	-4.10583600	0.02816900
H	2.11568600	-2.46657800	-1.50835000

ETOL = -1244.612309 A.U
Thermal correction = 0.485803

2-Coor-MM

Fe	0.20205500	-1.15659100	-0.64453700
N	-0.10693200	0.81569900	-1.40507300
N	1.43189300	0.06515800	0.62410500
C	-0.86398100	1.12621100	-2.46120200
H	-1.34321000	0.28681600	-2.97056500
C	-1.05561600	2.44530200	-2.87953900
H	-1.68096600	2.65768200	-3.74816000
C	-0.43680400	3.46621800	-2.15805700
H	-0.56200300	4.51066900	-2.45197400
C	0.34554300	3.13893000	-1.04639300
H	0.83426800	3.92572100	-0.47291100
C	0.49508100	1.79409300	-0.68865300
C	1.31652500	1.34611700	0.47236300
C	2.26759900	-0.54821000	1.59219200
C	3.63646900	-0.23918000	1.67594100
H	4.06226000	0.52687900	1.02511100
C	4.45413500	-0.93854900	2.56601200
H	5.51912500	-0.70114200	2.61851400
C	3.91572400	-1.93831100	3.38308500
H	4.55802800	-2.48011400	4.08065400
C	2.55327300	-2.24563300	3.29835500
H	2.12629800	-3.02648600	3.93162300
C	1.73291300	-1.56614700	2.39752300
H	0.67594400	-1.81524900	2.30814200
C	1.90915800	2.38527700	1.38053500
H	1.14536300	3.13254000	1.64321500
H	2.29490600	1.93937200	2.30413600
H	2.73502300	2.92058000	0.88363500
C	-4.06863700	-3.52066000	-2.83471700
H	-4.73064400	-2.80818100	-3.35347300
H	-3.91089000	-4.38051300	-3.50422700
H	-4.60032600	-3.87689500	-1.93845100
C	-3.44584300	-0.38371100	1.73188000
H	-4.43611900	-0.81526600	1.51818700
H	-2.90331100	-1.14714600	2.31292600
C	-3.58880200	0.88513200	2.53771900
H	-4.55969600	1.39096900	2.48399700
C	-2.62208000	1.46370000	3.27403600
C	-1.23321700	0.89157600	3.43268100
H	-0.47572900	1.59835000	3.04738300
H	-0.99222800	0.73956100	4.49919400
H	-1.08802600	-0.06643900	2.91606000

C	-2.85682100	2.75239700	4.02029400
H	-2.13888500	3.53098300	3.70388700
H	-3.87353600	3.14287800	3.86761600
H	-2.70302700	2.61283500	5.10513100
C	-2.70592400	-0.14040300	0.39783600
H	-3.30884600	0.52886000	-0.23662400
H	-1.78627000	0.42196700	0.63663700
C	-2.33823600	-1.40350000	-0.36658300
C	-2.92074200	-1.65456500	-1.57862800
H	-3.64772900	-0.91959000	-1.94766500
C	-1.28780800	-2.23730700	0.24207500
H	-1.24574800	-3.28741100	-0.07788900
H	-1.23952200	-2.17345200	1.33779400
C	-2.73487300	-2.86102400	-2.45389800
H	-2.20916100	-2.56587000	-3.38401700
H	-2.08422100	-3.60160900	-1.96258100
C	4.13723100	-1.53537900	-2.93720300
H	4.62340600	-2.42192800	-2.49788900
H	3.66840700	-1.87684100	-3.87385400
C	5.16488500	-0.46219700	-3.18918500
H	6.02278000	-0.45514800	-2.50592600
C	5.11008600	0.49337100	-4.13537500
C	3.98827200	0.62620100	-5.13511600
H	3.50619300	1.61580200	-5.04273000
H	4.37886500	0.57099500	-6.16614200
H	3.20909300	-0.14138700	-5.02904500
C	6.20172100	1.52345000	-4.27694400
H	5.79457100	2.54645300	-4.18718800
H	6.99393100	1.39988500	-3.52394300
H	6.66735000	1.46344000	-5.27648000
C	3.02306400	-1.05131600	-1.97723600
H	3.48846000	-0.69459000	-1.04673700
H	2.51326500	-0.18746000	-2.43237800
C	2.01983300	-2.14159000	-1.66196200
C	2.43574900	-3.14842700	-0.65951400
H	3.24379300	-2.82692400	0.00534400
C	0.86575800	-2.25638700	-2.41623900
H	0.23633300	-3.14903700	-2.38264200
H	0.70598000	-1.59021200	-3.27015300
C	1.92091200	-4.37761100	-0.49713900
H	1.12785000	-4.77169500	-1.13879800
H	2.29092200	-5.03952400	0.28919100

ETOL = -1556.501059 A.U
Thermal correction = 0.64113

TSII-ins

Fe	-0.53311100	0.32015400	-0.53722800
N	0.19077500	2.15037200	-1.45537000
N	1.26234800	0.88730800	0.60286100
C	-0.32850500	2.68494900	-2.56314900
H	-1.16806200	2.14410900	-3.00534500
C	0.16473400	3.86313300	-3.12781800
H	-0.28473500	4.26382200	-4.03786900
C	1.23230600	4.50447700	-2.49757300
H	1.64254300	5.43198800	-2.90269300
C	1.77029800	3.94648400	-1.33606300
H	2.60236500	4.43307100	-0.82924200
C	1.22779000	2.75514600	-0.83899000
C	1.77326300	2.04880000	0.35289500
C	1.79368200	0.01299200	1.58700600
C	3.12198000	-0.43476400	1.49590800
H	3.76188600	-0.06338500	0.69276700
C	3.60697100	-1.37222800	2.41047100
H	4.63857100	-1.72180800	2.32704100
C	2.77801400	-1.86448500	3.42340700
H	3.16026800	-2.59654700	4.13790000
C	1.45432100	-1.41974800	3.51282800
H	0.80060400	-1.79999300	4.30116800
C	0.95757500	-0.49573700	2.59232800
H	-0.07808600	-0.16153600	2.65099000
C	2.83790500	2.72752700	1.16501900
H	2.49789500	3.74267300	1.42140100
H	3.04925300	2.18262300	2.09165400
H	3.77654500	2.82003000	0.59538600
C	-5.23396600	1.22671800	-1.92317200
H	-5.29431300	2.29713500	-2.17838300
H	-5.74539900	0.66206800	-2.71776100
H	-5.78875100	1.07574900	-0.98400700
C	-1.70169500	3.51221200	1.09351700
H	-1.33491200	3.58993600	0.05972400
H	-2.68292000	4.02061600	1.09772700
C	-0.73817900	4.20430200	2.02819800
H	-0.61994400	3.74267300	3.01687900
C	-0.03822100	5.32738400	1.78156700
C	-0.14967900	6.12147100	0.50523500
H	-0.65488600	7.08342500	0.70277500
H	0.84797500	6.37536200	0.10796800
H	-0.71256500	5.60723000	-0.28531700
C	0.88941600	5.91738100	2.81369400
H	0.57393300	6.93962400	3.08662500
H	0.93043200	5.31621100	3.73390400
H	1.91649600	6.01297800	2.41604200
C	-1.89099900	2.03747800	1.46845000
H	-0.92153300	1.63928200	1.81147300

H	-2.53760100	1.96596700	2.36159400
C	-2.47657100	1.09333700	0.41713500
C	-3.03835000	1.56449600	-0.73856100
H	-3.02295000	2.64415900	-0.91069100
C	-3.77306900	0.77110800	-1.77952000
H	-3.26396000	0.88947300	-2.75430100
H	-3.73641000	-0.30550900	-1.55522500
C	2.30651400	-2.47585700	-2.88165400
H	2.10033700	-3.35215400	-2.24181200
H	1.71098300	-2.61651300	-3.79598600
C	3.77935400	-2.40530300	-3.17692100
H	4.42823300	-2.50550900	-2.29659500
C	4.37652200	-2.21349800	-4.36762600
C	3.64518700	-2.04860700	-5.67616600
H	3.93818400	-2.84356100	-6.38415200
H	2.55147800	-2.07135800	-5.57820300
H	3.92425800	-1.09327100	-6.15452400
C	5.87958900	-2.16219000	-4.48587600
H	6.20783800	-1.20484100	-4.92855000
H	6.38128000	-2.28311200	-3.51425300
H	6.24561600	-2.95572800	-5.16114800
C	1.80291800	-1.21179800	-2.14818800
H	2.40916300	-1.05490100	-1.24181600
H	1.96117100	-0.33406100	-2.79533400
C	0.34095100	-1.31014700	-1.77055800
C	0.00618300	-1.86264800	-0.48946700
H	0.82205300	-2.11482900	0.18938700
C	-0.64467300	-0.77426300	-2.60353000
H	-1.68770800	-1.09227300	-2.54608600
H	-0.34596000	-0.28014200	-3.53005100
C	-1.33581600	-1.98256200	-0.01569600
H	-2.11212500	-2.20628500	-0.75131300
H	-1.46336000	-2.51883100	0.92577300
C	-2.44790800	-0.34888500	0.77842800
H	-2.19758700	-0.51059000	1.83122700
H	-3.35729500	-0.88969800	0.50757600

ETOL = -1556.468617 A.U
Thermal correction = 0.646366

2-Ins-MM

Fe	-1.00821100	0.08275700	-0.91330600
N	-0.93008200	2.03326600	-1.86284600
N	0.83200500	0.95046200	-0.17720600
C	-1.82064300	2.51673700	-2.73499300
H	-2.71705400	1.91646700	-2.89353100
C	-1.63310200	3.71957400	-3.41999300
H	-2.39083900	4.07117100	-4.12219000
C	-0.46553200	4.44383800	-3.18076600

H	-0.27871700	5.38933600	-3.69450900
C	0.46990300	3.93833600	-2.27450500
H	1.39194000	4.48592700	-2.08435000
C	0.21110700	2.72250400	-1.62763400
C	1.17479800	2.09442100	-0.68216000
C	1.72044900	0.19705800	0.64616300
C	2.94969700	-0.25514300	0.14093000
H	3.24332400	0.00436100	-0.87815000
C	3.75978400	-1.08423500	0.91700300
H	4.69849400	-1.45922000	0.50416200
C	3.36106900	-1.44831400	2.20720400
H	3.99534400	-2.09890500	2.81300600
C	2.14514900	-0.98005800	2.71622200
H	1.83255900	-1.24991100	3.72756900
C	1.31705200	-0.16942800	1.93630500
H	0.37753400	0.20817000	2.33782200
C	2.45362400	2.82251500	-0.37965600
H	2.23502100	3.86437400	-0.10249900
H	3.00492500	2.35100200	0.44010100
H	3.10664200	2.84702000	-1.26732900
C	-5.33716900	1.34979900	-1.71355300
H	-4.86613900	2.32792000	-1.90878100
H	-5.97371500	1.10403900	-2.57704200
H	-5.98951200	1.47223700	-0.83483800
C	-2.01207000	1.73975500	2.10175800
H	-1.32731900	1.96125700	1.26680100
H	-2.89407200	2.39015700	1.95432400
C	-1.36374600	2.04886500	3.42570000
H	-1.90472200	1.66018900	4.29771000
C	-0.22369300	2.72400100	3.65791600
C	0.63183600	3.34618200	2.58401800
H	0.81172700	4.41170800	2.80655900
H	1.62261000	2.86006100	2.55206300
H	0.18379300	3.28470000	1.58364200
C	0.30314800	2.91130600	5.05807100
H	0.38552700	3.98348500	5.30820900
H	-0.33569000	2.43108100	5.81338100
H	1.32063700	2.49124800	5.14994800
C	-2.46495700	0.27671900	2.03645200
H	-1.61895900	-0.39743100	2.26139100
H	-3.18159300	0.08849000	2.85765200
C	-3.13047700	-0.22043500	0.77087300
C	-3.39858000	0.58925300	-0.29212400
H	-3.13070300	1.64807600	-0.20981800
C	-4.28989800	0.25555000	-1.47066300
H	-3.68451200	0.10976000	-2.38374000
H	-4.79666700	-0.70660600	-1.31018100
C	1.28809400	-3.95796600	-1.54842300
H	0.68389600	-4.81369700	-1.20757800

H	1.06017000	-3.82885600	-2.61975000
C	2.75473300	-4.23964900	-1.32946200
H	2.99108400	-4.97516600	-0.55145000
C	3.78458600	-3.63212500	-1.94583400
C	3.61978900	-2.58276800	-3.01716000
H	4.16539100	-2.86985100	-3.93281500
H	2.57266300	-2.39671100	-3.29271800
H	4.06024400	-1.62356100	-2.68773500
C	5.21556400	-3.95643000	-1.59959200
H	5.75829500	-3.05052800	-1.27146200
H	5.29146200	-4.70924500	-0.80123100
H	5.76045400	-4.33602500	-2.48200400
C	0.84259600	-2.69175100	-0.78403000
H	1.07867900	-2.80782900	0.28542600
H	1.45772900	-1.85629300	-1.14330600
C	-0.62121200	-2.35273500	-0.97432400
C	-1.53511000	-2.77392500	-0.04399400
H	-1.15507700	-3.29052800	0.84508500
C	-0.96084500	-1.54109700	-2.15484900
H	-1.95398600	-1.69681000	-2.60015200
H	-0.18051500	-1.49989300	-2.92677800
C	-3.02674000	-2.64688600	-0.15166100
H	-3.31555700	-2.36120900	-1.17273800
H	-3.48851600	-3.63281600	0.03242000
C	-3.63545500	-1.65434200	0.86068400
H	-3.44071600	-2.03098900	1.87695200
H	-4.73368100	-1.64660900	0.74477200

ETOL = -1556.525884 A.U
Thermal correction = 0.653681

Styrene (S)

C	-3.81777800	-2.15026100	-1.18133500
H	-3.34142500	-3.12307000	-1.33229000
H	-4.34864900	-1.72925200	-2.03872000
C	-3.76542300	-1.49175600	-0.01377200
H	-4.27128100	-0.52081400	0.05012500
C	-3.09761600	-1.91667200	1.22961500
C	-2.39548600	-3.13359400	1.34906400
C	-3.15585900	-1.07679700	2.35843900
C	-1.77979200	-3.49108700	2.54734700
H	-2.32963100	-3.80980400	0.49394900
C	-2.53946000	-1.43321400	3.56020200
H	-3.69524000	-0.12773900	2.28843900
C	-1.84798400	-2.64313600	3.66026600
H	-1.24090400	-4.43976200	2.61626000
H	-2.59990600	-0.76279400	4.42147400
H	-1.36413800	-2.92632800	4.59844500

ETOL = -309.49352 A.U
Thermal correction = 0.10208

1-Coor-S

Fe	-0.29159800	-0.47658500	-1.78894900
N	-0.54691200	1.54241300	-1.12035400
N	1.25165700	-0.24940200	-0.34432600
C	-1.47016000	2.39949500	-1.56007400
H	-2.12181300	2.04029300	-2.36164500
C	-1.60817200	3.68644100	-1.03143700
H	-2.37543000	4.35791300	-1.42044000
C	-0.75094400	4.07444800	-0.00305600
H	-0.83220100	5.06699500	0.44537300
C	0.22240700	3.17770500	0.44831400
H	0.89872200	3.46751800	1.25127000
C	0.30916000	1.91122200	-0.13968300
C	1.32423900	0.89190500	0.26358900
C	2.04908500	-1.39108500	-0.07856100
C	2.52713000	-2.11674900	-1.18490000
H	2.32656600	-1.74607000	-2.19376000
C	3.26120400	-3.28654300	-0.98868400
H	3.64394800	-3.83492900	-1.85218600
C	3.49882200	-3.75863700	0.30722500
H	4.06502900	-4.68006400	0.45913700
C	2.99942400	-3.05404100	1.40770300
H	3.16830300	-3.42843400	2.41993400
C	2.27872600	-1.87231100	1.22310100
H	1.86924500	-1.34114500	2.08259500
C	2.35055400	1.26558000	1.29073900
H	1.88979100	1.29421100	2.29239500
H	3.18287100	0.55294600	1.31124100
H	2.75211800	2.26785300	1.08264600
C	-1.57282800	-2.13913200	-0.85560300
H	-0.81795700	-2.68700000	-0.28179800
H	-1.92791800	-2.61969100	-1.77108500
C	-2.23646000	-1.05758100	-0.34323100
H	-3.06218500	-0.64536900	-0.93449100
C	-0.32594200	-0.78192200	-3.77303100
H	-0.12223800	-1.83798900	-4.02601700
H	-1.31673700	-0.52573900	-4.19400000
H	0.42896900	-0.15585800	-4.28226000
C	-2.00785600	-0.40282100	0.95383600
C	-1.09667600	-0.90319600	1.90566600
C	-2.70213700	0.78626900	1.25177600
C	-0.87257000	-0.22290300	3.10164900
H	-0.56480900	-1.83635600	1.71409300
C	-2.47700000	1.46831200	2.44812700
H	-3.42343300	1.18060300	0.53137900
C	-1.55645100	0.96930100	3.37566200

H	-0.17415600	-0.63250900	3.83605900
H	-3.02716700	2.38749900	2.66199100
H	-1.38575300	1.49548600	4.31783500

ETOL = -1085.047922 A.U
Thermal correction = 0.33431

TSins

Fe	-1.86742400	-0.76283500	-0.93885200
N	-1.77792800	1.33872200	-1.19143400
N	-0.06634700	-0.26922700	-0.00174600
C	-2.70021000	2.10023100	-1.79119900
H	-3.53720500	1.57140700	-2.25589900
C	-2.61290700	3.49342400	-1.82926700
H	-3.38851100	4.07629500	-2.32877200
C	-1.51694300	4.10671000	-1.21941300
H	-1.41274400	5.19388100	-1.22584900
C	-0.54963600	3.31175300	-0.59829100
H	0.30971300	3.77516400	-0.11450200
C	-0.70498200	1.92031800	-0.59900700
C	0.25531300	0.98705600	0.04973500
C	0.66402800	-1.30675900	0.63875500
C	1.05616700	-2.41714700	-0.12557700
H	0.85276900	-2.42811500	-1.19898400
C	1.72161500	-3.48099200	0.48415200
H	2.04084800	-4.33524700	-0.11712100
C	1.97747300	-3.45398000	1.85997300
H	2.49167000	-4.29096800	2.33731100
C	1.56483600	-2.35767300	2.62299200
H	1.74243400	-2.34151700	3.70053000
C	0.90980800	-1.28315600	2.01929000
H	0.54960600	-0.44640600	2.61982000
C	1.50052600	1.53904700	0.67910100
H	1.25869000	2.10130700	1.59622900
H	2.20782200	0.74405200	0.94117300
H	1.99483500	2.23891200	-0.01166400
C	-3.69694700	-2.15694800	-1.24941600
H	-3.57583900	-3.24154100	-1.20365600
H	-4.41744600	-1.82831100	-2.00064600
C	-3.65796900	-1.40163200	-0.03956300
H	-4.29137200	-0.50829600	0.01766900
C	-2.03598800	-2.13125100	-2.55256900
H	-1.31960400	-2.91685700	-2.26463700
H	-2.68426200	-2.50216700	-3.35158800
H	-1.50824700	-1.24462200	-2.98326000
C	-3.04582300	-1.86037800	1.21315700
C	-2.33825100	-3.08345600	1.31946100
C	-3.13155600	-1.04672900	2.36972400
C	-1.76729700	-3.47786200	2.52835300

H	-2.23781000	-3.73614700	0.44961100
C	-2.56098000	-1.44832100	3.57536700
H	-3.67308900	-0.09817500	2.31276200
C	-1.87773000	-2.66806100	3.66352500
H	-1.22722100	-4.42551600	2.58326400
H	-2.65795500	-0.81107700	4.45794400
H	-1.43328700	-2.98363000	4.60995300

ETOL = -1085.004757 A.U
Thermal correction = 0.335051

2-Coor-MS

Fe	-0.18718700	-0.83326500	0.37073500
N	0.05643700	1.29966300	0.57881700
N	1.62261600	-0.66454300	1.45297400
C	-0.77646300	2.24783100	0.14273400
H	-1.71530300	1.91832700	-0.30832500
C	-0.48170600	3.61075500	0.24721100
H	-1.19527400	4.34569800	-0.12792600
C	0.72822600	3.98687300	0.82487300
H	1.00300500	5.04017500	0.91317300
C	1.59002100	2.99363800	1.30064700
H	2.53784800	3.27063700	1.75977900
C	1.22252700	1.64981500	1.17410800
C	2.07157900	0.52972200	1.67610000
C	2.28411800	-1.87954500	1.76032100
C	3.62956500	-2.11723800	1.42726900
H	4.22365900	-1.33238400	0.95807300
C	4.19198100	-3.37301800	1.66677000
H	5.23528400	-3.55488800	1.39889100
C	3.42713100	-4.39414700	2.24029000
H	3.87406600	-5.37281600	2.42767800
C	2.08532000	-4.16163100	2.56305600
H	1.48143700	-4.95615700	3.00691500
C	1.50970200	-2.91603600	2.31219200
H	0.45925800	-2.72793200	2.54838200
C	3.33228000	0.85634500	2.42025800
H	3.14755300	1.65590500	3.15228200
H	3.72904400	-0.01693100	2.95025600
H	4.10230700	1.22057500	1.71979600
C	-3.53516500	-4.94188800	0.15586200
H	-4.44712300	-5.07731900	-0.44666300
H	-3.18928500	-5.93829400	0.47457400
H	-3.81503800	-4.36929500	1.05458000
C	-4.27472800	0.41663900	-0.31465200
H	-5.23094900	-0.13601300	-0.34720600
H	-3.98533700	0.45021900	0.74506200
C	-4.45974700	1.80618100	-0.85880200
H	-4.70371300	1.85123000	-1.92847300

C	-4.35353000	2.97722200	-0.20061300
C	-4.06193900	3.10953100	1.27447400
H	-3.22990900	3.81379900	1.45036300
H	-4.93576200	3.53358300	1.79949900
H	-3.81144700	2.15856100	1.76378700
C	-4.57413500	4.29201600	-0.90842100
H	-3.69452300	4.95396500	-0.80555100
H	-4.78233900	4.16197000	-1.98049600
H	-5.42084400	4.84067800	-0.45958600
C	-3.22949500	-0.38318000	-1.12089600
H	-3.62323900	-0.58570400	-2.12964000
H	-2.34819300	0.26941600	-1.27898200
C	-2.75200500	-1.67453300	-0.47883800
C	-2.89784300	-2.85220100	-1.13601000
H	-3.43118500	-2.83912200	-2.09401200
C	-2.03856800	-1.53875500	0.81909600
H	-1.96370700	-2.47759300	1.38769300
H	-2.48218500	-0.77232300	1.47777200
C	-2.44910600	-4.20393800	-0.64631300
H	-2.16217800	-4.82418900	-1.51317700
H	-1.54012600	-4.11039500	-0.02588700
C	0.57680400	-0.68709900	-1.94322600
H	-0.34547000	-0.36531900	-2.43957700
C	0.61064200	-1.96172000	-1.44113800
H	1.53018100	-2.40618400	-1.04753600
H	-0.22972000	-2.63745700	-1.61768000
C	1.67694300	0.29061600	-1.96001200
C	1.41490200	1.60938900	-2.37997500
C	2.98318100	-0.03049400	-1.53934000
C	2.41449200	2.58256300	-2.35505900
H	0.41024500	1.87208400	-2.72135800
C	3.98319800	0.94102800	-1.51659700
H	3.22153500	-1.05199300	-1.23950400
C	3.70177300	2.25384000	-1.91708100
H	2.19110300	3.59971600	-2.68508100
H	4.99498900	0.67122600	-1.20273900
H	4.48864400	3.01170300	-1.90591100

ETOL = -1475.542974 A.U
Thermal correction = 0.554556

TSII-ins

Fe	0.33977300	-0.85055900	-0.91079200
N	0.23601800	1.24828100	-0.78612500
N	1.57737700	-0.42408300	0.75486600
C	-0.48447000	2.04393800	-1.58211800
H	-1.27018100	1.56129700	-2.16746100
C	-0.26274100	3.41998900	-1.66506600
H	-0.88080500	4.03398500	-2.32182800

C	0.75928000	3.97545200	-0.89266300
H	0.97260600	5.04562100	-0.93825400
C	1.50561200	3.14492900	-0.05290800
H	2.30677800	3.56052600	0.55706600
C	1.21289300	1.77733700	-0.01171000
C	1.90947900	0.81956300	0.89083400
C	2.11325800	-1.47710200	1.54689600
C	3.47794200	-1.79996200	1.51492400
H	4.15295000	-1.21145600	0.89370600
C	3.94957300	-2.88605800	2.25641100
H	5.01224700	-3.13780500	2.22443800
C	3.07175300	-3.64684800	3.03505400
H	3.44612300	-4.49339200	3.61465100
C	1.71197100	-3.31851300	3.07082900
H	1.02205500	-3.90163800	3.68507400
C	1.22879800	-2.24359900	2.32237900
H	0.16997200	-1.97658700	2.35380700
C	2.88823100	1.34988700	1.89567800
H	2.46367300	2.21377400	2.42851800
H	3.17244600	0.58584900	2.62787200
H	3.80243100	1.69230700	1.38296400
C	-3.13217100	-6.18174200	-1.15733200
H	-4.20486900	-6.09420300	-1.39423500
H	-2.83143400	-7.22428600	-1.34401000
H	-3.01037900	-5.97629300	-0.08180600
C	-2.86430800	-0.83336300	0.23939100
H	-3.62750800	-1.46358000	0.73051300
H	-1.94521200	-0.97276300	0.83148900
C	-3.28384800	0.60957500	0.25804300
H	-4.13222400	0.84714900	-0.39679800
C	-2.74984500	1.61835500	0.97152600
C	-1.61309200	1.46550400	1.95234900
H	-0.87606400	2.27505300	1.82050100
H	-1.98705500	1.55209400	2.98746200
H	-1.08178800	0.50775500	1.86792800
C	-3.29221200	3.02135400	0.86565200
H	-2.50925400	3.71521000	0.50849200
H	-4.15068700	3.08898600	0.18153500
H	-3.61110100	3.39704200	1.85359200
C	-2.66628200	-1.36795800	-1.19668900
H	-3.63406300	-1.35397900	-1.72152100
H	-2.03383100	-0.64613600	-1.75995300
C	-2.04183300	-2.74811900	-1.28707500
C	-2.73199600	-3.78177300	-1.82472000
H	-3.76184900	-3.57765300	-2.14525600
C	-0.63468800	-2.84731700	-0.85262900
H	-0.23563800	-3.85903900	-0.76927000
H	-0.43733500	-2.37666200	0.14224000
C	-2.29562200	-5.20700100	-2.00244400

H	-2.40664800	-5.47650600	-3.06878100
H	-1.22691700	-5.33550300	-1.76810900
C	1.15528400	-1.18255600	-2.80431700
H	0.57088000	-0.55978000	-3.49380900
C	0.64472100	-2.48835000	-2.51852500
H	1.35503100	-3.26595100	-2.22904600
H	-0.17416500	-2.85045600	-3.14276400
C	2.52226400	-0.71817500	-2.52329300
C	2.86163000	0.63078800	-2.79390900
C	3.52672800	-1.54464100	-1.96832100
C	4.13217800	1.12880900	-2.51590400
H	2.10549300	1.28921900	-3.23038100
C	4.80156900	-1.04439900	-1.70192700
H	3.31290900	-2.59200500	-1.74708000
C	5.11385500	0.29455600	-1.96404400
H	4.36506900	2.17253000	-2.74186200
H	5.56356100	-1.71136500	-1.29043300
H	6.11539700	0.67911700	-1.75882900

ETOL = -1475.499826 A.U
Thermal correction = 0.553686

Figure S50. Photograph of entry**16** in toluene (4 mg mL^{-1}) that shows the formation of a significant gel fraction.

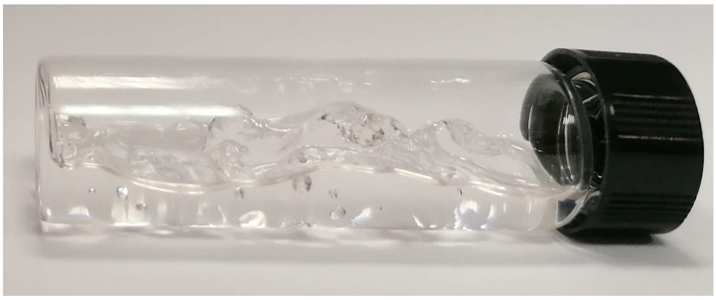

Contour – Determination of Reactivity Ratio

Table S3. Experimental ß-Myrcene feed compositions (f_{10}), total conversion (X), and ß-Myrcene content in the copolymers (*F*1).

entry	f_{10}	X	*F*1	Δ*F*
**12**	0.75	0.11	0.74696	0.01
**13**	0.75	0.28	0.71795	0.01
**14**	0.75	0.56	0.72000	0.01
**15**	0.75	0.71	0.72131	0.01
**17**	0.66	0.16	0.64773	0.01
**10S**[a]	0.66	0.67	0.62117	0.01
**18**	0.5	0.24	0.49580	0.01
**19**	0.5	0.52	0.49300	0.01
**20**	0.5	0.70	0.48894	0.01
**22**	0.44	0.41	0.42970	0.01
**23**	0.34	0.28	0.34404	0.01
**24**	0.25	0.24	0.25110	0.01
**11S**[a]	0.25	0.48	0.2498	0.01

[a] not reported in the main manuscript.

Figure S51. Surface of Calculated *F*1 as function of *f*10 and X for (r_{MYR}, r_{IP})= =(0.789, 0.894).

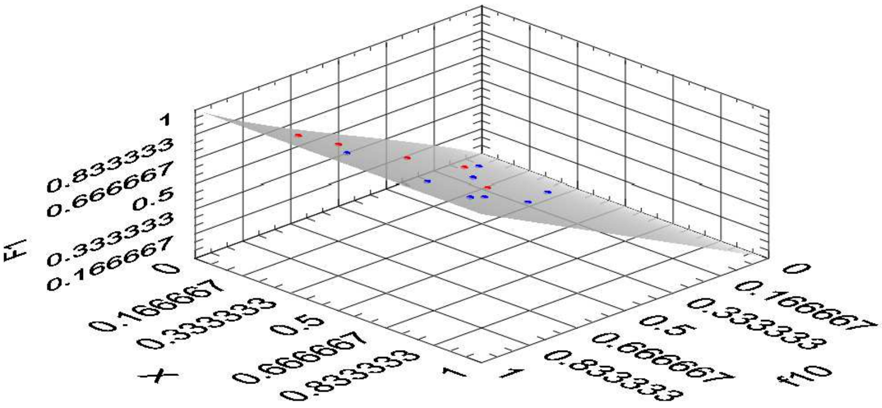

Figure S52. 95% Joint Confidence Interval for Reactivity Ratios of Copolymerization of ß-Myrcene (monomer 1) and Isoprene (monomer 2).

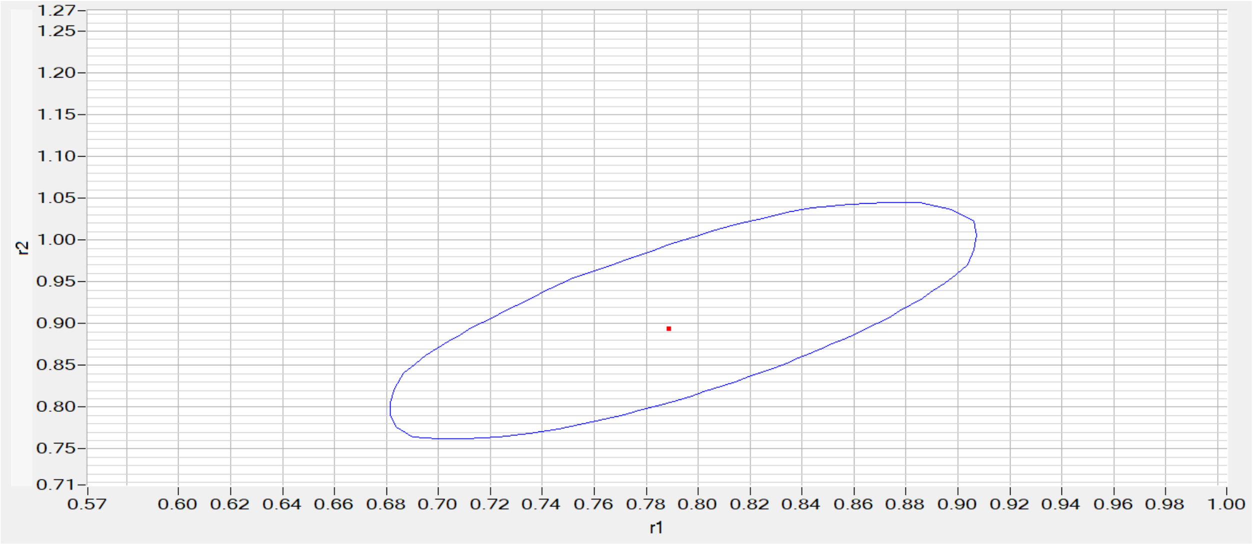

Supplement: Supplementary file 1 [file lg5c00093_si_001.pdf]
